# Efficient Preconstruction of Three-Dimensional Graphene Networks for Thermally Conductive Polymer Composites

**DOI:** 10.1007/s40820-022-00878-6

**Published:** 2022-06-14

**Authors:** Hao-Yu Zhao, Ming-Yuan Yu, Ji Liu, Xiaofeng Li, Peng Min, Zhong-Zhen Yu

**Affiliations:** 1 Beijing Key Laboratory of Advanced Functional Polymer Composites, Beijing University of Chemical Technology, Beijing, 100029 People’s Republic of China; 2 College of Materials Science and Engineering, Beijing University of Chemical Technology, Beijing, 100029 People’s Republic of China; 3 School of Chemistry, CRANN and AMBER, Trinity College Dublin, Dublin, Ireland

**Keywords:** Graphene networks, Thermal conductivity, Thermal interface materials, Phase change composites, Anisotropic aerogels

## Abstract

Electronic devices generate heat during operation and require efficient thermal management to extend the lifetime and prevent performance degradation. Featured by its exceptional thermal conductivity, graphene is an ideal functional filler for fabricating thermally conductive polymer composites to provide efficient thermal management. Extensive studies have been focusing on constructing graphene networks in polymer composites to achieve high thermal conductivities. Compared with conventional composite fabrications by directly mixing graphene with polymers, preconstruction of three-dimensional graphene networks followed by backfilling polymers represents a promising way to produce composites with higher performances, enabling high manufacturing flexibility and controllability. In this review, we first summarize the factors that affect thermal conductivity of graphene composites and strategies for fabricating highly thermally conductive graphene/polymer composites. Subsequently, we give the reasoning behind using preconstructed three-dimensional graphene networks for fabricating thermally conductive polymer composites and highlight their potential applications. Finally, our insight into the existing bottlenecks and opportunities is provided for developing preconstructed porous architectures of graphene and their thermally conductive composites.

## Highlights

- Fundamental principles for designing high-performance thermally conductive graphene-based polymer composites are reviewed in detail.
- The reasoning behind using the preconstructed graphene 3D networks for fabricating thermally conductive composites and recent progress are discussed in-depth.
- Insight into the existing bottlenecks and opportunities in developing preconstructed 3D networks of graphene and their thermally conductive composites is also presented.

## Graphical abstract

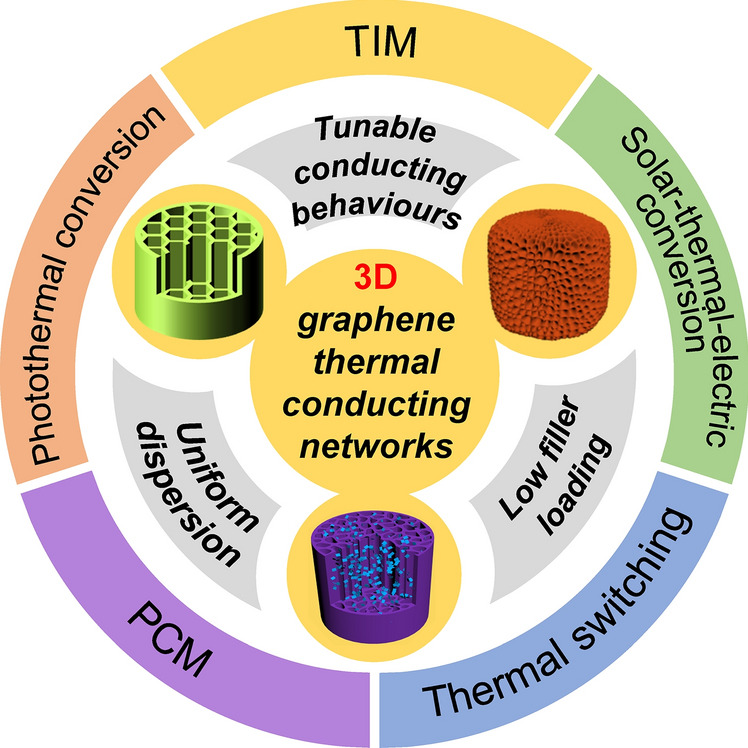

## Introduction

With the fast development of powerful electronic devices, high-performance thermally conductive materials that can provide efficient thermal management are becoming increasingly important and attracting more and more attention. Among all the thermally conductive materials, polymer composites have significant advantages because of their lightweight, easy processability, low cost, and superb stability. Most polymers are typically insulative with extremely low thermal conductivity of 0.1–0.3 W m^−1^ K^−1^ [1]. Thus, incorporating thermally conductive fillers into polymers represents a cost-effective and efficient way to fabricate thermally conductive materials that can combine high thermal conduction of functional fillers and superior properties of polymers. Since its discovery in 2004 [2], graphene has revolutionized the field of thermally conductive materials because of its extraordinary in-plane thermal conductivity [3] and superb mechanical properties [4, 5]. The unique ultrathin two-dimensional (2D) structure of graphene with ultrahigh aspect ratios makes it an ideal functional filler for polymer composites to achieve desirable thermal conductivities. To date, graphene/polymer composites have found widespread use in electronics [6, 7], communication equipment [8, 9], and energy harvesting, conversion and storage [10–13].

Typically, the thermal conduction and other properties (e.g., mechanical performances) of graphene/polymer composites are largely determined by the dispersion and distribution of graphene sheets in the polymer matrix. To achieve efficient thermal transport in polymer composites, highly interconnected graphene networks should be formed. Conventional methods to fabricate thermally conductive graphene/polymer composites rely mainly on directly mixing the graphene with polymers by in situ polymerization [14, 15], solution processing [16–18], and melt compounding [19]. Although these methods provide simple and scalable routes for the fabrication of composites, there exist two significant issues. One is the aggregation of graphene sheets during the mixing process. As a result, thermally conductive networks in polymer matrices can only be formed at relatively high graphene loadings, which would result in limited thermal conductivities, high cost, and degraded mechanical properties of polymer composites. The other one is that the graphene distribution and the configuration of as-formed conducting networks cannot be effectively tuned during simple blending processes and the graphene sheets typically show random dispersion and distribution in the resulting composites, which result in monotonous function and severely limit wide applications of the composites.

Recently, the preconstruction of three-dimensional (3D) continuous networks of graphene sheets followed by backfilling polymers has been proved to be a very effective method to fabricate composites with improved performances. The preformed conducting networks can be well maintained during subsequent compounding with polymers, avoiding aggregation of graphene sheets in polymer matrices and hence enabling high thermal conductivities at relatively low graphene loadings while preventing mechanical performance degradation of polymers [20, 21]. More importantly, the thermally conductive behaviors of the resultant composites can be tuned by configurational/microstructural designs of the preconstructed 3D graphene networks, such as designing anisotropic thermally conductive networks for enabling directional conducting behavior, which provides unique flexibility and versatility for composite fabrication and would significantly broaden the application of the as-fabricated composites.

Although great progress has been made, hitherto, the evolution of using preconstructed 3D networks of graphene sheets for fabricating thermally conductive polymer composites has not been discussed in depth and recent progress has also not been well recognized and analyzed. To the best of our knowledge, there remains a lack of comprehensive summaries and guidance on how preconstructed graphene 3D conducting networks can be designed for functionalizing polymers facing thermal management applications. To this end, the present review aims to provide a focused and critical review on thermally conductive graphene/polymer composites and highlight the recent advancements in designing novel composites with preconstructed graphene networks as fillers. The characteristics of this review include: (1) the key factors that affect the thermal conductivity of graphene/polymer composites and strategies for achieving high thermally conductive properties are reviewed in detail; (2) the reasoning behind using the preconstructed 3D graphene networks for fabricating thermally conductive polymer composites is discussed in-depth; (3) the potential applications toward thermal managements of the graphene/polymer composites, such as thermal interface materials (TIMs), phase change materials (PCMs), photothermal conversion materials, and thermal switches, are demonstrated; and (4) the challenges and perspectives regarding the development of preconstructed graphene 3D networks and their thermally conductive polymer composites are also presented.

## Factors Affecting Thermal Conductivity of Composites Functionalized with 3D Graphene Networks

**Fig. 1:**
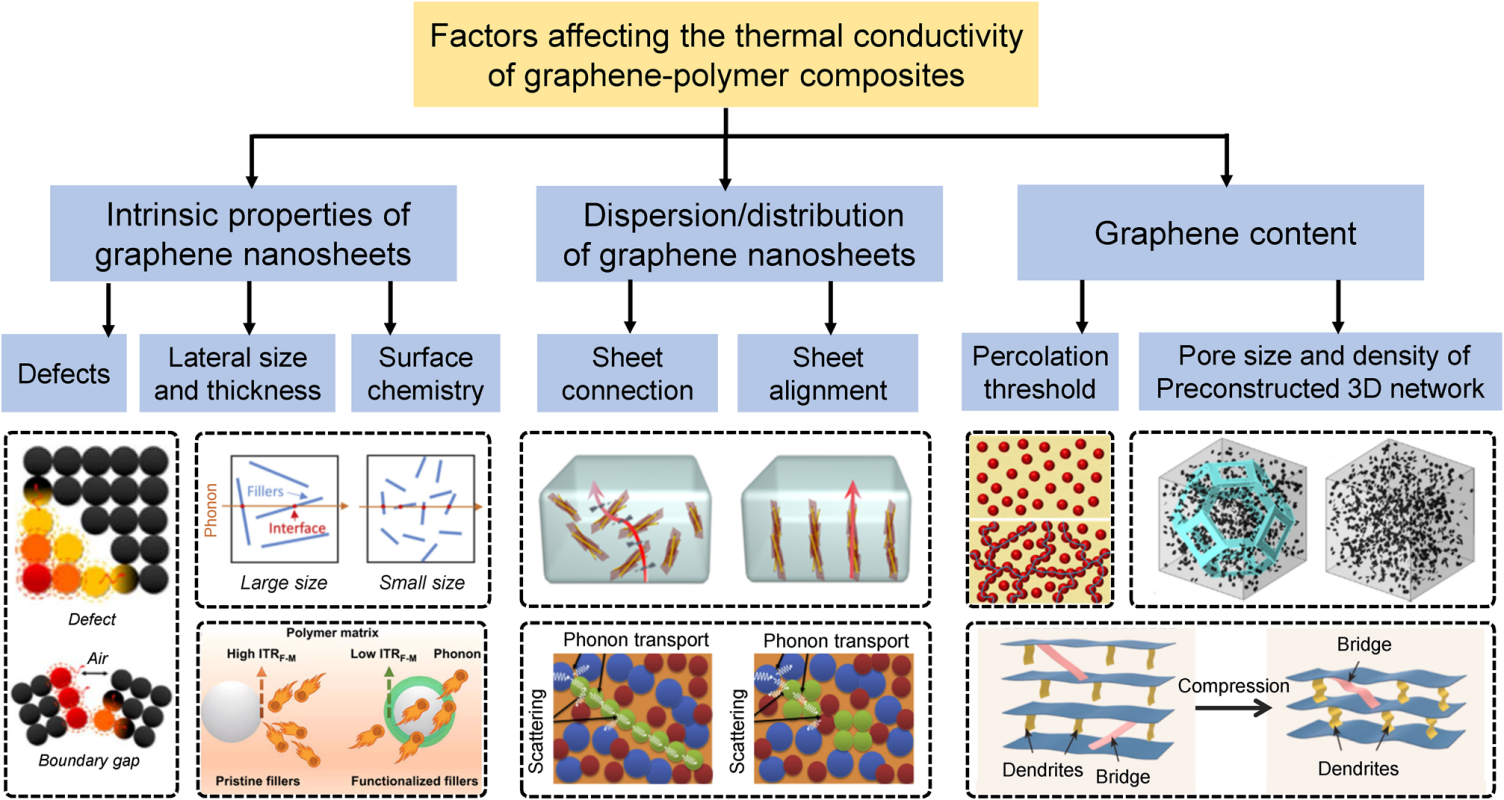
Factors affecting thermal conductivity of polymer composites functionalized by graphene 3D networks [22–29]

Focusing on polymer composites with graphene as fillers, the main factors that influence the thermal conductivities include: (1) intrinsic properties of graphene in terms of sheet quality, lateral size, thickness (layer number), aspect ratio, and interfacial compatibility with polymer matrix; (2) dispersion and distribution of the graphene in polymer composites; and (3) graphene loading level (Fig. 1).

### Effects of Intrinsic Properties of Graphene Sheets on Thermal Conductivity of Composites

**Fig. 2:**
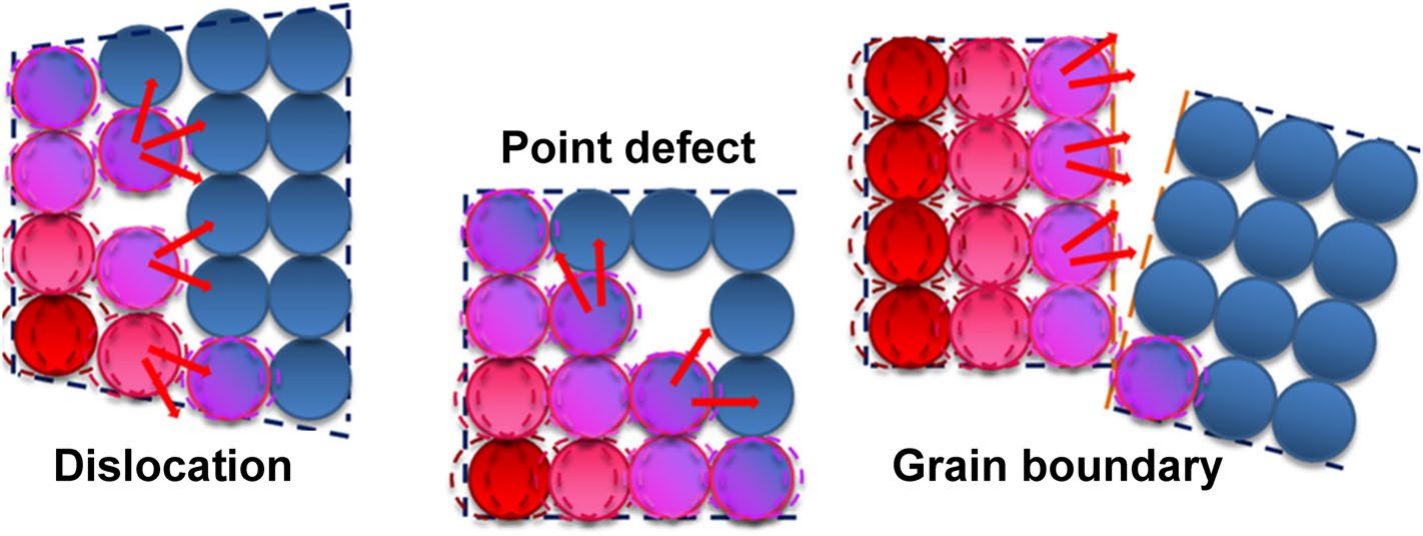
Schematic illustrations of phonon scattering in crystalline materials caused by defects [37]

The strong covalent sp^2^ bonding between carbon atoms as well as the unique 2D crystal structure that can restrain phonon scattering during heat transfer endow graphene with ultrahigh thermal conductivity. The heat in graphene sheets is predominantly transferred through phonon vibrations [30–32]. Previous studies have revealed that the existence of atomic defects can significantly reduce the thermal conductivity of graphene [33]. Usually, 3D graphene conductive networks can be obtained by assembling of graphene oxide (GO) building blocks followed by chemical/thermal reduction. However, the graphene sheets derived from mildly reduced GO often present large defect density and residual functional groups on the surface, which would shorten the long phonon mean free path of graphene by phonon scattering and cause heat loss, leading to degradation in thermal conduction (Fig. 2) [34–37]. Among all the reduction methods, high-temperature annealing of GO has been considered an effective approach in largely removing the oxygen functionalities and healing the lattice defects, ensuring high thermal conductivity [38, 39]. For instance, Li et al. reported that thermal annealing of graphene aerogel (GA) at 2800 °C increased the thermal conductivity of the GA/epoxy composite from 1.63 to 6.57 W m^−1^ K^−1^ [40]. An et al. reported that the thermal conductivity of epoxy composites enhanced by graphene foam (GF) increased with the annealing temperature of the GF, and the epoxy composite containing 2800 °C-annealed GF showed a superb thermal conductivity of 35.5 W m^−1^ K^−1^ with a graphene loading of 19.0 vol% [41].

**Fig. 3:**
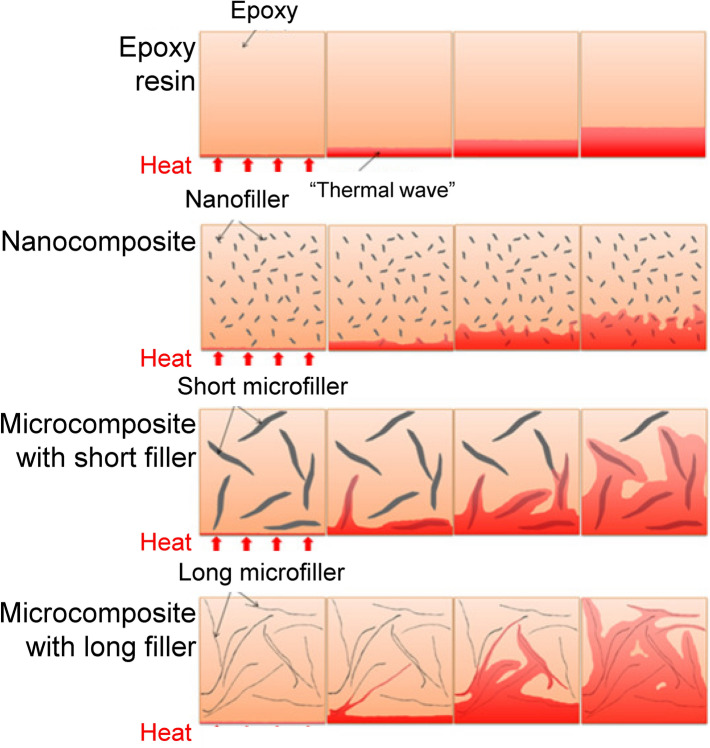
Schematic illustrations showing the heat transfer in epoxy composites containing fillers with different sizes [37]

The lateral size and thickness (layer number) of graphene sheets also significantly affect thermal conductivity of the as-formed networks. Theoretically, increasing lateral size is beneficial for enhancing thermal conductivity of graphene sheets and hence performances of composites since the sheet edge can scatter phonons (Fig. 3) [37, 42, 43]. It has also been noted that using fillers with smaller sizes would generate more interfacial area between the filler and polymer and lead to higher surface energy, making it more difficult to achieve uniform dispersion. On the basis of the simulation results reported by Su et al., in the length range of 0–16,000 nm, the thermal conductivity of infinite wide graphene nanoribbons increased with the length and eventually tended to be constant, and the thermal conductivity of graphene nanoribbons also increased with the width [44]. Hitherto, substantial efforts have been made to fabricate high-performance graphene materials by using large graphene sheets [38, 45, 46]. Besides, sheet thickness is another factor that affects thermal conductivity of graphene by interlayer phonon coupling effect. Ghosh et al. reported that the thermal conductivity drastically decreased from 2800 W m^−1^ K^−1^ for 2-layer graphene to 1300 W m^−1^ K^−1^ for 4-layer graphene [47].

Compared with high-quality graphene sheets, multilayer graphene nanoplatelet (GNP) with large lateral size and high aspect ratio is a cost-effective alternative for fabricating thermally conductive polymer composites [48]. In the network formed with these nanoplatelets, the thermal conductivity enhancement is achieved due to the longer phonon mean free path, lower interfacial density, and larger contact area between the nanosheets, which could ensure minimal phonon scattering and heat loss [49, 50]. Shen et al. reported that the epoxy-based composite made from multilayer graphite nanoplatelets with large aspect ratios possessed thermal conductivity even higher than those containing monolayer or few-layer graphene at the same graphene loading [51]. Note that, for situations where ultra-high performances of the materials are required, the use of ultrathin and large graphene sheets is still preferred despite the cumbersome procedure and high cost for the synthesis processes.

**Fig. 4:**
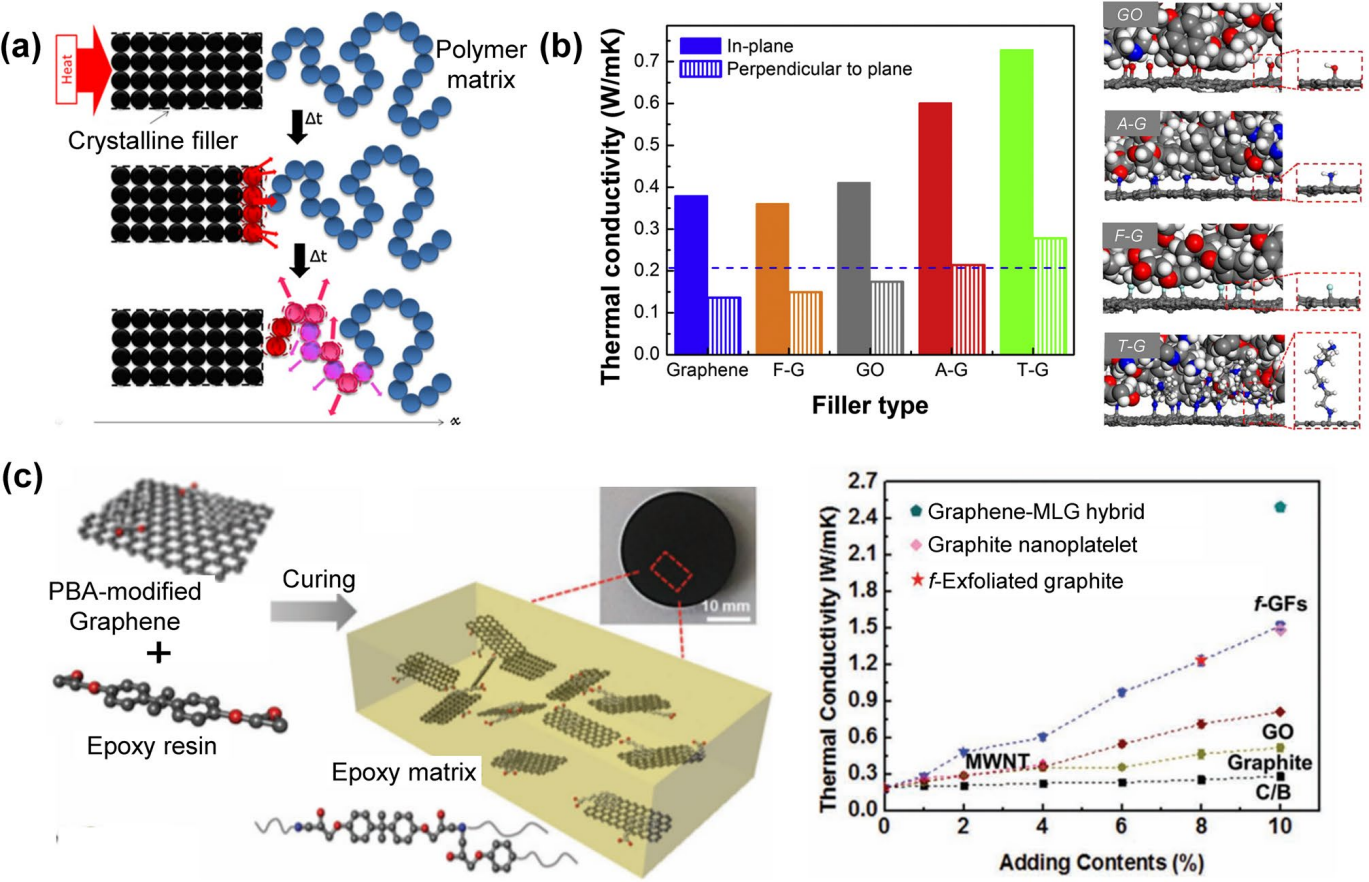
**a** Schematic illustrating the thermal conduction mechanism at the interface between the crystal filler and the polymer [37]. **b, c** Enhancement of thermal conductivity of graphene/polymer composites by graphene surface modification [23, 54]

For graphene/polymer composites, the large thermal resistance caused by phonon scattering at the interface between graphene sheets and matrix would hinder the efficient heat transfer seriously [52]. To reduce the interfacial thermal resistance for further increasing the thermal conductivity of the composite, surface modification of graphene sheets has been used to improve interfacial compatibility between graphene and matrices, as schematically shown in Fig. 4 [23, 37, 53, 54]. For example, the functional groups on ethylenediamine-reduced graphene aerogel (EGA) could alter the hybrid and vibration modes and increase the vibration coupling degree between carbon atoms of graphene and tetradecanol (TD) matrix, which could effectively reduce the energy loss at the interface during heat transfer. The TD-based composite made from EGA exhibited a thermal conductivity of up to 1.092 W m^−1^ K^−1^ at a graphene loading of 10 wt% [55]. Covalent grafting of polyamide 6 (PA6) chains onto reduced graphene oxide (RGO) sheets by in situ thermal polycondensation could improve the interfacial compatibility and reduce the contact thermal resistance between PA6 matrix and graphene. After the modification, the thermal conductivity of the GF/PA6 composite was improved to 0.847 from 0.210 W m^−1^ K^−1^ of neat PA6 [56]. It should be noted that the surface modification is more effective for graphene with small sheet sizes. This is because small graphene sheets in the polymer would generate more filler-matrix interfaces, which significantly hinder the phonon transportation, and the improved interfacial compatibility and enhanced thermal transfer could compensate for the reduction in the intrinsic thermal conductivity of the graphene sheets after surface modification. When the size of graphene increases, fewer filler-matrix interfaces can be formed and the quality of graphene would become the dominant factor for determining the thermal conductivity. In this case, the surface modification that decreases the intrinsic conductivity of graphene sheets would lead to significant degradation in thermal conductivity of the composites. Therefore, a critical lateral size exists for surface-modified graphene, above which the positive effect of surface modification for graphene would be suppressed. Note that the critical size is dependent on the functionalization types, matrix types and filler content [23].

### Effects of Ordered Dispersion and Distribution of Graphene Sheets on Thermal Conductivity of Composites

Directional thermally conductive networks, which can provide highly effective heat transfer pathways and lead to reduced percolation threshold along specific directions, can be formed by controlling the alignment/orientation of graphene sheets in the composites. Various methods have been reported to tune the aligning behavior of graphene in polymer matrix with the aid of external forces, electric fields, and magnetic fields. For example, the controllable alignment of graphene fillers in polymers can be achieved by modifying the graphene with magnetic particles and adjusting their orientation by an external magnetic field during the processing process, and the thermal conductivity of the graphene/epoxy composite made by this method is twice higher than that of the composite with randomly dispersed graphene sheets [57–59]. Despite the great progress, it is challenging to effectively tune the orientation while achieving homogeneous dispersion of graphene sheets in polymers by conventional processing methods, such as solution mixing, and melt compounding. Functionalizing polymer materials with pre-created graphene network with designed microstructures represents a promising way to allow for efficient control of graphene distribution in polymers. For example, Lian et al. prepared an epoxy composite by using a pre-created vertically aligned and interconnected graphene network, and the resultant composite exhibited excellent anisotropic thermal conductivity of 2.13 W m^−1^ K^−1^ along the vertical direction and 0.63 W m^−1^ K^−1^ along the horizontal direction at an ultralow graphene content of 0.92 vol%. Evidently, graphene sheets can largely overlap with each other to provide highly continuous conducting pathways along the vertical direction and the thermally insulating epoxy resin that fills in the oriented graphene channels can effectively suppress the heat transfer along the horizontal direction, thus enabling the unique anisotropic properties of the composite [60]. Other efforts have also been made to tune the thermally conductive behaviors along the horizontal direction by creating hierarchical bridging structures or designing radially oriented structures [61, 62].

### Effect of Graphene Contents on Thermal Conductivity of Composites

The thermal conductivities of graphene/polymer composites defer to the percolation theory, and the content of graphene is the decisive factor determining the thermal conductivity of polymer composites [63, 64]. A higher graphene content means more contact between the sheets and more heat transfer pathways in the composite, thus ensuring higher thermal conductivity [65–67]. In conventional compounding methods by directly mixing graphene with polymers, high graphene contents usually cause the formation of agglomerates/clusters, which hinders the enhancement in thermal conductivity. This problem can be alleviated by preconstructing the conductive graphene networks [68, 69], which can ensure homogeneous distribution of graphene sheets in matrices and thus efficient enhancement in thermal conductivity. For example, Liu et al. preconstructed a graphene network, and its epoxy composite exhibited a thermal conductivity of 20.0 W m^−1^ K^−1^ at a low graphene loading of 4.28 wt% [70]. On the other hand, the graphene content can be easily tuned by adjusting the structure details of the preconstructed 3D graphene networks. Notably, ultrahigh graphene content and homogeneous dispersion can be simultaneously realized by reducing the pore size and increasing the density of the preconstructed 3D graphene network, enabling significant improvement in the thermal conductivity of polymer composites. For example, Mu et al. tuned the pore size of GAs by applying different reducing agents in the hydrothermal process and a higher thermal conductivity of the composites is achieved by using the preconstructed graphene network with smaller pore sizes [55]. Qi et al. synthesized a dense graphene network by a modified CVD method and demonstrated that the thermal conductivity of corresponding paraffin wax (PW) composite is 87% higher than that of the PW composite with a low-density graphene network prepared by an ordinary CVD method [71].

**Fig. 5:**
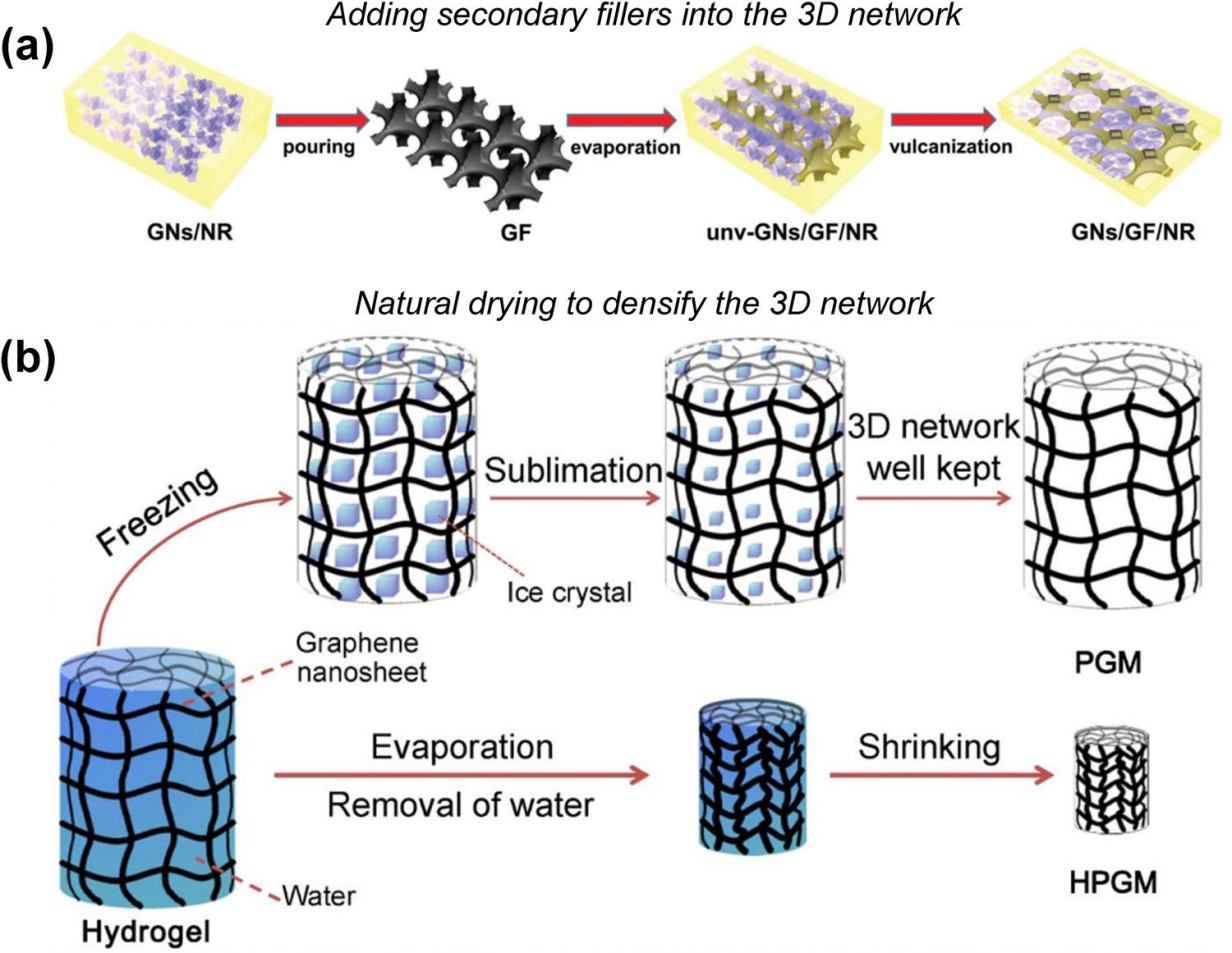
**a** Schematic illustration of GNs/GF/NR composites with compact network [75]. **b** Comparison of freeze-drying and air-drying [79]

Typically, most reported preconstructed graphene networks are obtained by CVD synthesis and freeze-drying/supercritical carbon dioxide (CO_2_) drying of their precursors including modified graphene suspension and RGO hydrogels. The insufficient interconnections between the graphene sheets in low-density graphene networks inevitably lead to large contact thermal resistance and low graphene concentration, limiting the enhancement in thermal conductivity of polymer composites [72–74]. To tackle this issue, Wu et al. filled the graphene network fabricated by CVD with graphene nanosheets (GNs) and natural rubber (NR) to increase the graphene loading in the composite (Fig. 5a) [75]. The pore size and density of graphene networks can also be tailored by controlling the drying conditions of graphene hydrogels (GHs) to achieve better continuity of graphene conduction networks and higher graphene loading in the composite. For example, natural drying of GHs under ambient conditions is a facile and cost-effective way to generate 3D graphene networks with smaller pore size, better continuity and higher density (Fig. 5b) [76–79]. Note that excessive volume shrinkage, even structural collapse, might occur during the natural drying process and hence the polymers cannot be well impregnated into the pores of GAs, causing poor performances of the composites [76, 79–81]. Undesired volume shrinkage can be suppressed by adding fillers that can support the graphene network, or by modifying the evaporation behavior of solvents. Yang et al. synthesized a high-density RGO/GNP aerogel by air-drying, in which the conductive GNPs not only prevent the excessive volume shrinkage but also enhance the thermal conductance of the RGO network, and its 1-octadecanol phase change composite exhibited an outstanding thermal conductivity of 5.92 W m^−1^ K^−1^ at a graphene loading of 12 wt% [77]. Li et al. found that the structural robustness of GHs can be well improved by forming a secondary polymer (e.g., polyacrylamide) network, ensuring high resistivity to structural collapse during vacuum-drying or air-drying processes, and the polymer can be removed easily by subsequent high-temperature annealing [82]. Xu et al. developed a natural drying strategy with a pre-freezing protocol, enabling the reduction in solvent evaporation capillary force and thus effectively inhibiting volume shrinkage during the drying process [83]. Another cost-effective method to generate high-density GAs is freeze-drying a concentrated putty-like GO paste (55–100 mg mL^−1^) followed by high-temperature annealing, by which a very high density of up to 100 mg cm^−3^ for the resulting GAs can be achieved [84].

In summary, the enhancement of the thermal conductivity for the composites is related to not only the quality and content of graphene but also the distribution and dispersion of graphene filler in the polymer matrix. Generally, heat transfer is realized through lattice vibrations within the material. Thus, to achieve satisfactory thermal conductivity of the composites, creating a highly continuous graphene network that allows for a high-speed heat transfer process in the polymer matrix is the most effective and convenient way, which can be achieved by proper processing/compounding processes.

## Constructing Graphene Networks in Composites by Blending Graphene Sheets with Polymers

Polymers are typically thermally insulative [37]. In graphene/polymer composites, the phonon scattering at the interface between graphene and polymer matrix hinders efficient heat transfer [85, 86], and the enhancement in thermal conductivity mainly relies on improving the interconnectivity of the graphene sheets. Therefore, to obtain composites with high thermal conductivity, a highly continuous thermally conductive network should be formed in polymers, in which the graphene sheets can be well dispersed and interconnected with each other to provide effective heat transfer pathways [87].

The thermally conductive network can be formed by directly dispersing the graphene sheets into polymer matrices by in situ polymerization, melt compounding, and solution mixing processes. These methods are very efficient and convenient for mass production. For example, Colonna et al. prepared graphene/poly(cyclic butylene terephthalate) (PCBT) composites by in situ ring-opening polymerization of cyclic butylene terephthalate with GNPs uniformly dispersed in the monomers [88]. Compared with the in situ polymerization method, directly blending polymers with graphene sheets is a more time-saving and scalable method to fabricate thermally conductive composites. Polymers can be dissolved in appropriate organic solvents or melted to possess flowability at elevated temperatures (for thermoplastic polymers, e.g., PA6, polypropylene (PP), and high-density polyethylene) [89], enabling favorable processability.

The challenge in the process of dispersing graphene sheets in polymers by the above-mentioned methods is the easy aggregation of graphene sheets during the blending processes as the van der Waals forces and *π*–*π* interactions between graphene sheets are stronger than the interactions between graphene and polymers. To tackle this issue, various methods, such as adding surfactants/additives and modifying the surface of graphene sheets are developed. The addition of surfactants can lower the surface tension between the solid graphene sheets and polymer liquids, which is effective in promoting the dispersion of graphene sheets and suitable for many polymer systems. The additives that possess good compatibility with both graphene and polymer matrices can be added during the compounding process. Chen et al. reported that the presence of GO in graphene/PA6 composite can improve the dispersion of graphene in PA6 as the GO can simultaneously interact with graphene by *π*–*π* interactions and with PA6 by forming covalent bonds [90]. Surface modification of graphene sheets relies on grafting functional groups or components that are compatible with polymers onto the graphene to facilitate the dispersion of graphene in the polymers [91]. For example, modifying graphene sheets with polydopamine (PDA) results in numerous hydrogen bonds between the modified-graphene and the polyvinyl alcohol (PVA) matrix, leading to a uniform dispersion of graphene in PVA and thus a more effective thermal conduction network in the composite [92]. However, it should be noted that the residual surfactants and additives in the composites might adversely affect their ultimate performances [93, 94].

Alternatively, the addition of second thermally conductive fillers with specific configurations is also effective for improving thermal conductivity of graphene/polymer composites on the basis of the synergistic effect between different conductive fillers [95]. For example, the synergistic effect of 2D graphene sheets and 0D Al_2_O_3_ particles can reduce the aggregation of graphene in polylactic acid (PLA) and the contact thermal resistance at the interface of fillers, resulting in an enhanced thermal conductivity of the graphene/Al_2_O_3_/PLA composite [96]. The synergistic effect between 2D graphene sheets and 1D conductive fillers can improve the interconnection between graphene sheets to enhance the continuity of the conductive network. For instance, the addition of 2 wt% multi-walled carbon nanotubes (MWCNTs) can increase the thermal conductivity of the graphene/polycarbonate composite by 23% as compared with the composite without the MWCNTs at the same graphene content [97]. Adding third organic or inorganic phases into graphene/polymer composites could allow graphene sheets to be selectively dispersed in polymer matrices outside the third phases, which is beneficial for forming more continuous graphene networks at lower filler loadings on the basis of the volume exclusion effect [98–103]. For example, the introduction of PLA into the graphene/polystyrene (PS) composite enables a selective dispersion of the graphene sheets in the PS phase, which significantly reduced the percolation threshold [104].

In summary, in the above-mentioned methods to fabricate thermally conductive graphene/polymer composites, the aggregation of graphene sheets cannot be effectively avoided during direct blending/compounding processes, and thus the efficient thermal conducting networks can usually be formed at high graphene loadings, resulting in limited thermal conductivities and even degraded mechanical properties of the composites. Moreover, the conventional fabrication methods suffer from poor controllability of the filler distribution and configuration of conducting graphene network in polymers, resulting in composites with monotonous functions.

## Preconstruction of Isotropic Graphene Networks and Their Thermally Conductive Composites

**Fig. 6:**
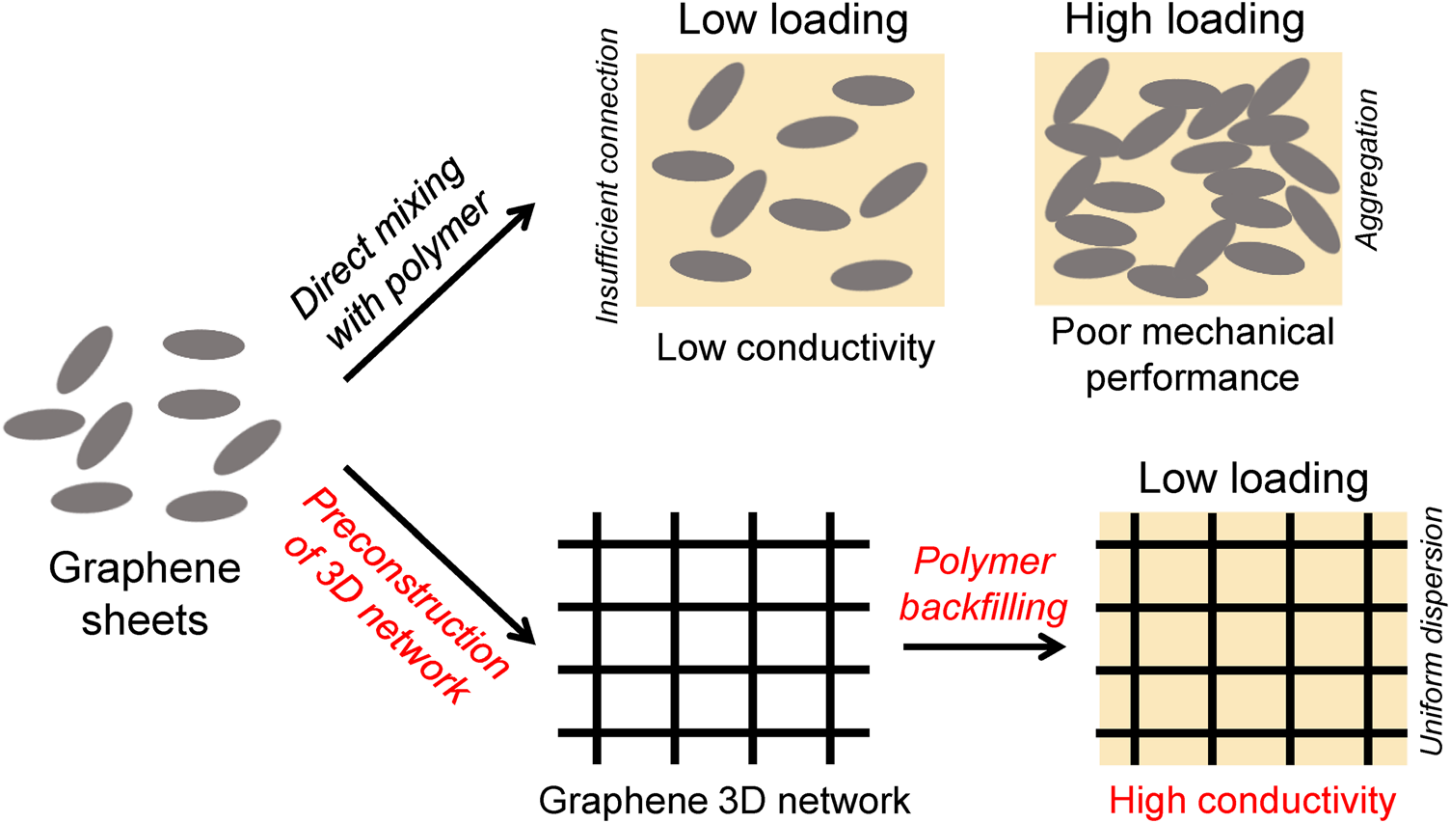
Schematic illustrating the graphene dispersion in polymer composites fabricated by different compounding methods

Preconstruction of 3D graphene networks followed by backfilling of polymers can enable the formation of efficient thermal conduction pathways and the fine control of graphene dispersion in composites, representing an effective way to fully utilize graphene for functionalizing polymers (Fig. 6). Because of the high continuity and integrity of the preconstructed 3D graphene networks in polymer matrices, outstanding thermal conductivities of the as-fabricated composites can be achieved even at ultralow graphene contents (Table 1). The isotropic graphene networks exhibit a disordered structure, in which thermal conduction is uniform in all directions. The typical fabrication strategies of isotropic graphene networks include self-assembly, the use of templates, and 3D printing method [105–107].

**Table 1:** Comparison of fabrication methods for thermally conductive graphene/polymer composites

Methods	Advantages	Disadvantages	Materials	Thermal conductivity (W m^−1^ K^−1^)	Filler loading	References
Solution mixing	Simple preparation process	Organic solvents are needed	GNP/PVA	13.4	10 wt%	[92]
	Low processing temperature	Aggregation of graphene sheets	GO/SR	0.34	0.1 wt%	[108]
	Easy incorporation of functional components		GNP/PVB	4.521	30 wt%	[109]
			Graphene/PE	1.84	10 wt%	[110]
			Graphene/PP	1.53	10 wt%	[110]
			Graphene/PVA	1.43	10 wt%	[110]
			Graphene/PVDF	1.47	10 wt%	[110]
			Graphene/BE	0.542	1.45 vol%	[111]
			GO-PDA/PS	4.56	0.96 vol%	[112]
Melt mixing	High productivity	High processing temperature	GNP/PC	1.13	20 wt%	[97]
	Simple preparation process	Decomposition of functional components				
		Restricted to thermoplastic materials	RGO/PA	5.1	5 wt%	[113]
		Aggregation of graphene sheets	GNP/PC	7.3	20 wt%	[50]
In-situ polymerization	Lower contact thermal resistance between graphene and polymer	Complex preparation process	Graphene/PA6	0.416	10 wt%	[14]
		Limited productivity	Graphene-MLG /Epoxy	5.1	10 vol%	[63]
			Graphene-GO/PA6	2.14	10 wt%	[90]
			f-G/PDMS	0.761	2 wt%	[114]
			GNPs/Epoxy	1.5	2.8 vol%	[51]
			GNP/PCBT	2.49	30 wt%	[88]
Pre-constructing 3D networks	Capability in fabricating highly conductive composites with ultralow filler content	Complex preparation process	GHF/Epoxy	35.5	19.0 vol%	[41]
	Uniform filler dispersion	Relatively high processing costs	DAGF/Epoxy	62.4	13.3 vol%	[115]
	Efficient control of distribution/alignment of fillers		GA/Epoxy	20	2.30 vol%	[70]
	Composites with novel performances can be fabricated		GNPs/GF/NR	10.64	5.78 vol%	[75]
			VAIGN/Epoxy	2.13	0.92 vol%	[60]
			GWFs/PI	3.73	12 wt%	[116]
			MGF/GF/PDMS	1.08	2.7 vol%	[28]
			GF/mGNPs/PVDF	6.32	9.07 vol%	[117]
			c-GA/MF/PEG	1.32	4.6 wt%	[118]
			Graphene/PA6	0.69	0.25 wt%	[119]
			GA/Epoxy	2.69	1.11 vol%	[120]
			GNPs/RGO/Epoxy	1.56	21.4 wt%	[9]
			GF/Epoxy	8.04	6.8 wt%	[121]

*SR* silicone rubber, *PVB* polyvinyl butyral, *PE* polyethylene, *BE* Bio-based polyester, *PC* polycarbonate, *PA* polyamide, *MLG* multilayer graphene, *f-G* functionalized grapheme, *PCBT* poly-cyclic-butylene terephthalate, *GHF* graphene hybrid foam, *DAGF* dual assembled graphene framework, *VAIGN* vertically aligned and interconnected graphene network, *GWF* graphene woven fabric, *MGF* multilayer graphene flake, *mGNP* modified graphene nanoplatelet, *c-GA* carbonized graphene aerogel

### Self-Assembly Method

**Fig. 7:**
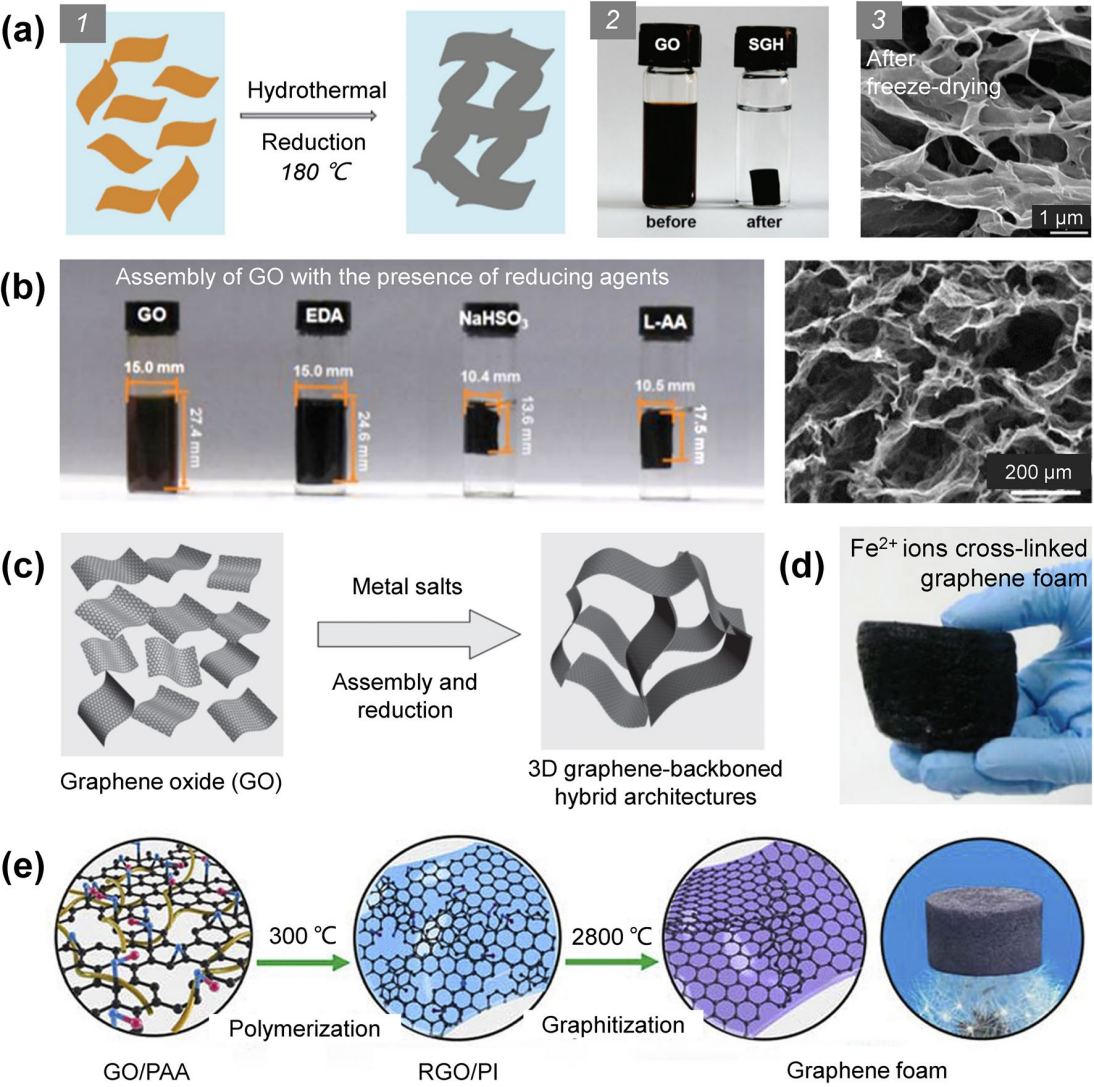
**a** Schematic of formation of networks during hydrothermal reduction in microscale, optical photographs of GO suspension and GHs formed by hydrothermal reduction and the scanning electron microscope (SEM) image of the graphene network [123]. **b** Optical photographs of GO suspension and GHs formed by chemical reduction and the SEM image of the graphene network [124]. **c–e** Schematics of the microscopic mechanism of the formation of graphene 3D networks induced by adding multivalent metal ions and water-soluble polymers and optical photographs of the graphene foams formed after post-treatment [125–127]

Fabrication of 3D graphene networks by self-assembly relies on forming bonding, cross-linking, or physical interactions between the graphene precursors (mainly GO and RGO), through which a balance between the electrostatic repulsive forces and the bonding interaction is achieved, ensuring the integrity of the 3D interconnected networks while preventing the excessive aggregation of the graphene components [122, 123]. The functional groups on the GO and RGO sheets endow them excellent solution processability, and the regulation of interactions between the sheets can be easily realized by partially removing the surface oxygenated groups with hydrothermal reduction and/or chemical reduction, enabling the formation of 3D graphene architectures by the self-assembly (Fig. 7) [123–127]. Hydrothermal treatment is an simple yet effective method to reduce GO in aqueous mediums at elevated temperature and pressure for forming 3D GHs [123, 128]. Similarly, chemical reduction relies on the reducing agents used, such as ethylenediamine (EDA) [129, 130], ascorbic acid (VC) [131, 132], ammonia [133] and hydrazine hydrate [134, 135]. Compared with hydrothermal method, chemical reduction allows for a faster reaction rate under lower temperatures or even ambient conditions [136]. Among all the reported reducing agents, VC can reduce the GO to induce a mild gelation and would not generate toxic gaseous products during the reduction process, resulting in uniform 3D networks [137]. To improve the manufacturing efficiency, a well-recognized treatment strategy has been widely used by adding reducing agents into hydrothermal systems [8, 131, 138]. For example, the addition of EDA into a dispersion of GO can significantly decrease the hydrothermal time and also introduce amino functional groups onto the RGO sheets to enhance the interlayer interactions, through which the as-obtained GA exhibited better structural robustness [138]. Tang et al. reported that paraphenylene diamine can facilitate the reduction in GO to form a 3D hydrogel and functionalize the surface of GO sheets by grafting reaction, which can effectively prevent the volume shrinkage of the 3D RGO structure during the hydrothermal process [72]. Zhang et al. prepared a GA by hydrothermal treatment of a GO hydrosol with the presence of EDA followed by freeze-drying and high-temperature annealing, and its silicone rubber composite presented an outstanding thermal conductivity of 1.26 W m^−1^ K^−1^ (448% enhancement) at an ultralow GA loading of 0.50 wt% [139]. Note that adding the additives (e.g., water-soluble polymers and multivalent metal ions) that can interact with the GO sheets into the precursor dispersion can also induce the gelation of GO sheets, leading to the formation of integrated graphene networks upon proper post-treatments including thermal annealing, hydrothermal synthesis, and freeze-drying (Fig. 7c–e) [125–127].

### Isotropic Template-Assisted Assembly Method

In the template method, isotropic graphene 3D networks are formed by growing/depositing graphene layers on the skeletons of porous templates via chemical vapor deposition (CVD) or impregnation with graphene dispersions, and the templates can be removed by post-treatments [140–142]. Popular templates include Ni foam for CVD method [143, 144] and polymer sponges (e.g., polyurethane (PU) sponge [145] and melamine sponge [146]) for the impregnation method.

**Fig. 8:**
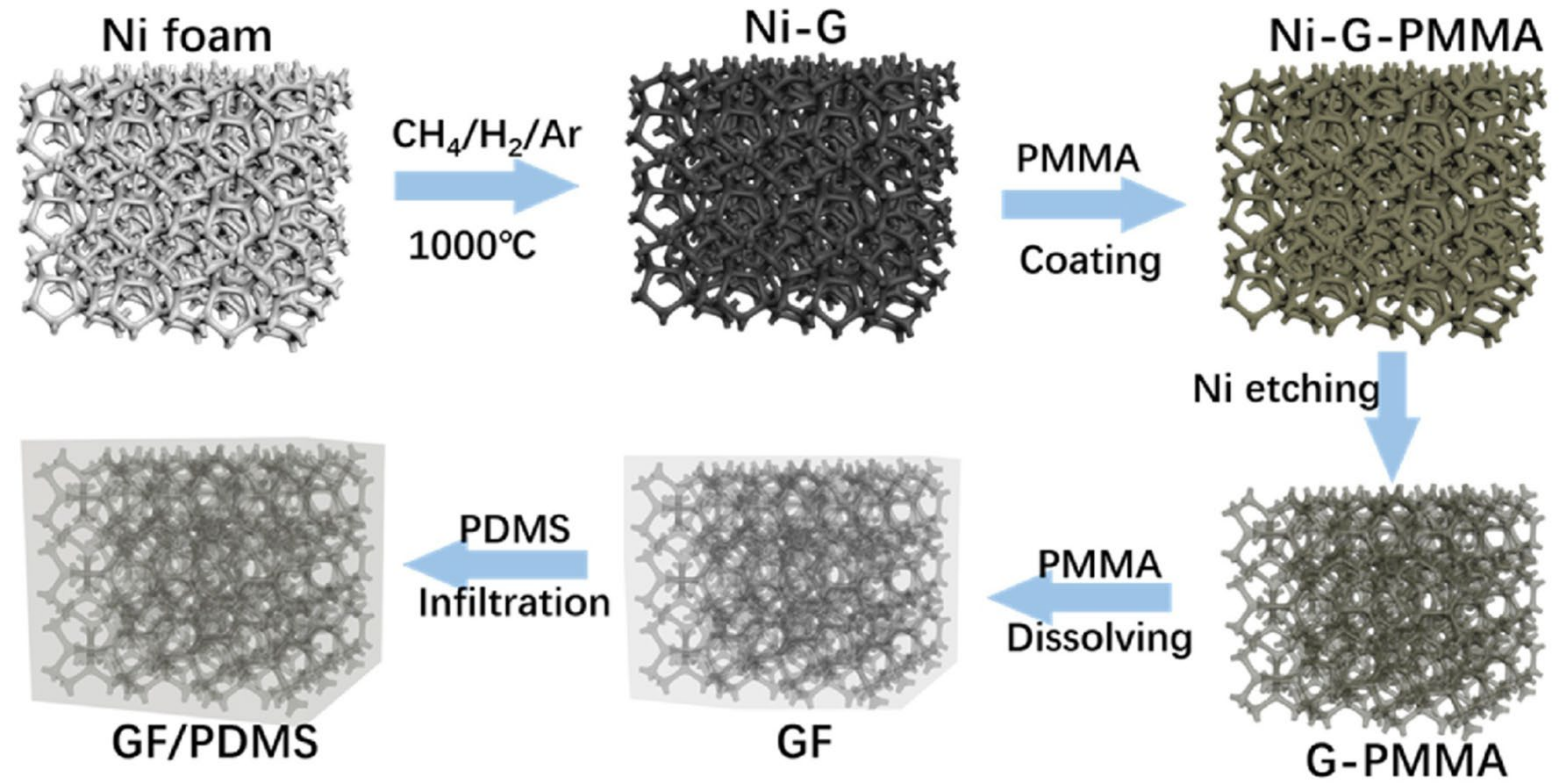
Schematic of synthesis of GF and the GF/PDMS composite by isotropic template-assisted assembly method [147]

Growing graphene on the template via a CVD method can generate 3D networks composed of ultra-thin and high-quality graphene layers, which typically possesses better thermal conduction than the network formed with RGO. However, the challenge of this method lies in preventing the structure collapse during the template removing process because the as-formed graphene networks are typically very brittle [140]. Great efforts have been made to solve the above-mentioned problems (Fig. 8) [147]. For example, Chen et al. coated a PMMA layer on a Ni/graphene foam to reinforce the structure before the Ni template etching process, which effectively prevents the structural collapse of graphene network, and the PMMA layer can be easily removed by acetone [140]. To date, this method has been widely used in enhancing the graphene network. Yang et al. combined the template method with self-assembly to fabricate 3D graphene networks. In a typical process, the Ni foams were first dipped into GO/GNP/VC mixture followed by hydrothermal treatment and freeze-drying to generate a hybrid graphene aerogel (HGA) inside the Ni foam template. Additional graphene was grown subsequently on the HGA/Ni foam via a CVD approach, and the freestanding GF/HGA can be obtained by removing the Ni foam template. The construction of the HGA structure in the Ni foam template can effectively suppress the massive expansion and destruction of the graphene network by gaseous products during the CVD process. Moreover, the HGA network also provides thermal conduction pathways to enhance the thermal conductivity, and its paraffin wax composite delivers a thermal conductivity of 1.82 W m^−1^ K^−1^ along with outstanding shape stability [148].

Besides, graphene networks can be obtained by depositing graphene sheets on the skeleton of polymer sponge templates by vacuum-assisted impregnation, and the templates can be removed by pyrolysis. For example, Liu et al. immersed a PU foam into a dispersion of graphene, and the foam was then dried after suction filtration or centrifugation. The surface of the PU foam skeleton was covered by a continuous graphene layer, and a freestanding graphene 3D network was obtained after the pyrolysis of the PU foam. In the above-mentioned work, the as-fabricated epoxy composite exhibited a thermal conductivity of 8.04 W m^−1^ K^−1^ at a graphene content of 6.8 wt%, which is 4473% higher than that of pure epoxy resin [121]. In another work by Xue et al., melamine foams (MFs) were used as the template, which were dipped into a GO/GNP/ VC dispersion followed by hydrothermal treatment to obtain hybrid hydrogels, and corresponding graphene networks were obtained by freeze-drying and carbonization. The PCM composed of such a hybrid graphene network and PW had a high thermal conductivity of 1.46 W m^−1^ K^−1^ at a filler content of 4.89 wt% [149]. In addition, mixing the polymeric microspheres with GO could enable the selected distribution of GO sheets in the gap between polymer spheres, providing an alternative strategy to generate highly continuous 3D graphene networks in polymer composites [150]. Meanwhile, the polymer microspheres as sacrificial hard templates can be easily removed by organic solvents to generate freestanding 3D graphene networks [151]. Although using polymer templates to induce the assembly of graphene sheets provides a cheaper and more scalable way to generate graphene networks than CVD method [121, 152], further efforts are required to realize high quality of the as-fabricated graphene networks, which is the key for achieving high thermal conduction [121, 152].

### 3D Printing Method

As a newly emerged manufacturing technology that promises high design freedom, 3D printing can be used to accurately produce objects with complex shapes/structures [153]. The outstanding solution processability of GO and RGO suspensions makes it possible to form viscous GO or RGO inks that can be printed to generate 3D structures, and corresponding graphene architectures can be obtained by post-treatments including freeze-drying and chemical/thermal reduction [154]. By designing the printing process, 3D graphene networks and corresponding functional composites can be fabricated easily on demand to meet requirements for diverse application scenarios [155–157].

Designing of highly printable inks with appropriate rheological properties is the key to applying 3D printing technique for generating 3D graphene architectures. In addition, post-treatment protocols, such as drying and thermal annealing after printing, should be carefully designed to convert the 3D printed wet materials to freestanding structures. The post-treatments can also largely determine the quality (e.g., shape fidelity, mechanical properties) of the printed devices. Ma et al. designed an aqueous GO ink, which presents favorable 3D printability even at a low solid concentration without requiring additional additives or freeze operations. With this ink, 3D graphene networks with desired configurations were easily fabricated via 3D printing followed by freeze-drying, chemical reduction, and thermal annealing [158]. Zhang et al. also reported an aqueous GO/EDA ink that can be injected into a 3D printed template, and a corresponding graphene hydrogel was obtained by hydrothermal treatment. By subsequent freeze-drying and thermal annealing, the solvents and polymer templates can be easily removed, resulting in freestanding cellular graphene networks [159]. Zhu et al. used fumed silica powder as a removable additive to modify rheological properties (e.g., viscosity, shear yield stress and shear thinning behavior) and printability of GO inks. An organic solvent bath with isooctane was used to effectively prevent the nozzle clogging and structural collapse during the printing process [160]. Besides, other GO-based ink modification strategies by adding polymers, like polyethylenimines and polyethylene glycol (PEG), were also reported, which can significantly increase the ink modulus and viscosity for 3D printing [161].

In summary, preconstruction of isotropic graphene 3D networks followed by compounding with polymers is a simple and effective strategy to create thermally conductive composites, through which significant enhancement of thermal conductivity can be easily realized even with very low graphene loadings. However, the resulting composites generally exhibit isotropic heat conductance and monotonous conducting behaviors, which limit their potentials for advanced thermal management applications.

## Preconstruction of Anisotropic Graphene Networks and Their Conductive Composites

The composites with anisotropic graphene networks can benefit from the anisotropic thermal conductivities of the conducting networks for enhancing heat transfer along with specific directions even at low graphene loadings. Due to the unique 2D layered structure and high intrinsic in-plane thermal conductivity of graphene sheets, their alignment can be controlled to form anisotropic graphene networks with high continuity along specific directions, providing highly efficient thermally conductive pathways [162, 163]. Compared with the composites with an isotropic graphene network, the anisotropic graphene/polymer composites can exhibit much higher thermal conductivity along selected directions at the same graphene loading because the continuity of the heat transfer pathways is less affected by the thermally insulating polymers [164–166]. This directional heat transfer capability enables a great potential for applications as TIMs and thermal spreading materials (TSMs) [167, 168]. Intuitively, successful preconstruction of anisotropic graphene networks relies on fine regulating the orientation of graphene sheets and achieving favorable robustness of the as-formed structure. Hitherto, various methods such as self-assembly, directional freeze-casting, and template method have been reported, all of which provide feasible routes for producing anisotropic graphene networks.

### Self-Assembly Method

Creating anisotropic graphene networks by self-assembly is a simple method that allows on-step synthesis of desirable structure without requiring complex processing procedures. However, it is quite challenging to control the alignment of graphene sheets during the assembly process, which requires rational compositional design of precursors. One of the most commonly used methods to achieve anisotropy of graphene structure is to form GO liquid crystals in precursor suspensions, and the orientation behavior of the graphene sheets in liquid crystals can be transferred to the ultimate macroscopic 3D network after the assembly process [169].

**Fig. 9:**
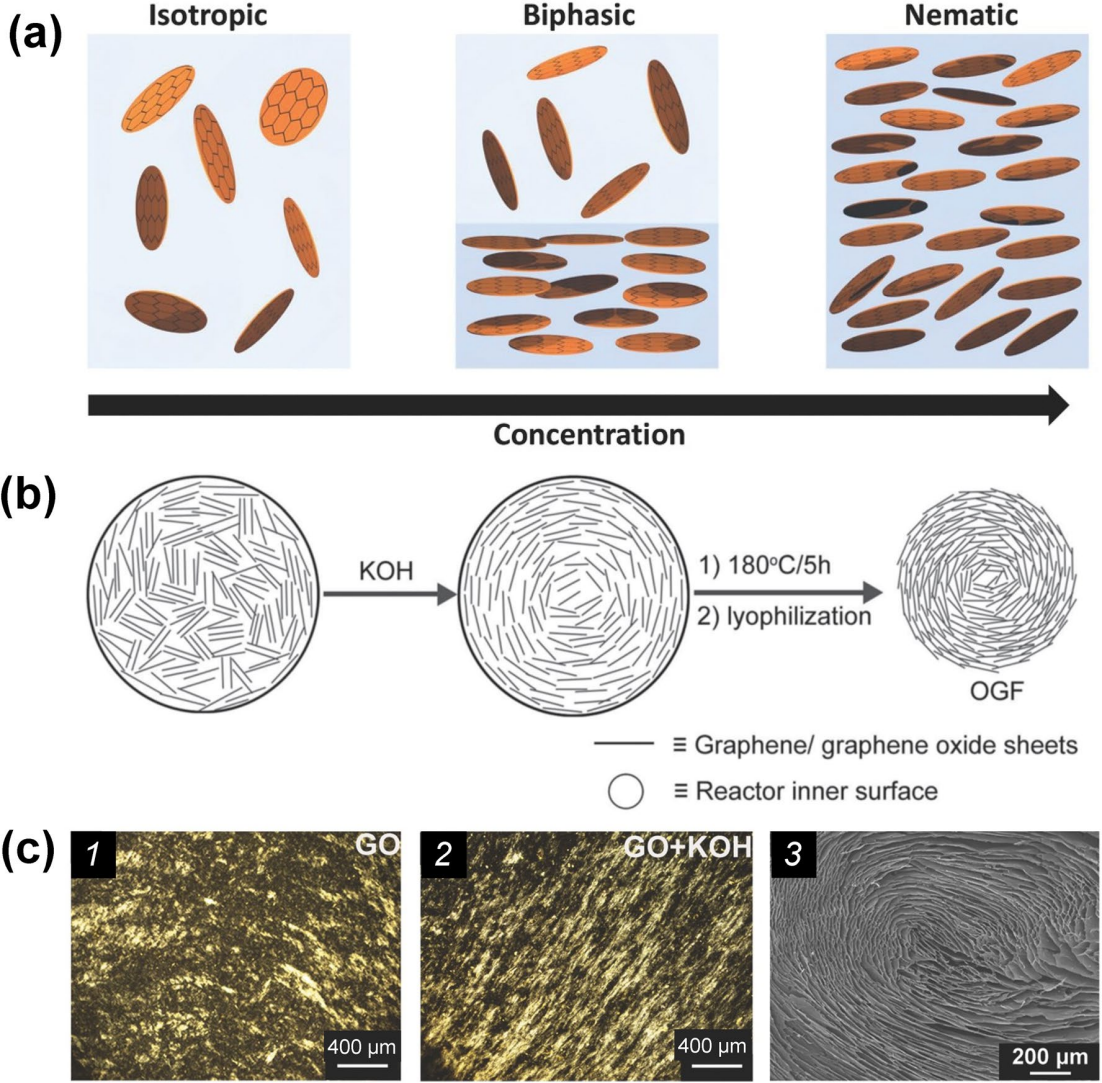
**a** Schematics of the phase transition of GO liquid crystal with the increase in concentration [171]. **b** Microscopic schematics of the formation of the anisotropic graphene 3D network by the orientation of GO liquid crystal [175]. **c** Polarized-light optical microscope and SEM images of the anisotropic graphene 3D network formed by the orientation of GO liquid crystal [175]

According to Onsager’s theory, 2D sheets in dispersions can form liquid crystals once the concentration exceeds a critical value [170, 171]. Compared with pristine graphene that has very limited solubility/dispersibility either in water or organic solvents, GO has outstanding dispersibility and thus the capability for forming liquid crystals because of its rich surface chemistry. With increasing the GO concentration, there is an isotropic-nematic phase transition of GO sheets (Fig. 9a) [171], and the nematic phase of GO liquid crystals could be transformed into layered phase [172–174]. Note that the reduction of GO sheets that removes the oxygenated groups and extends the rigid domains can also contribute to the formation of the liquid crystals in the dispersion and then facilitate the formation of an anisotropic structure in the resulting 3D network [175]. Based on this principle, controlling the reduction of GO to induce the formation of liquid crystals during the self-assembly process represents a promising way to prepare anisotropic graphene networks [175]. Wang et al., fabricated anisotropic GAs using HI-assisted hydrothermal treatment followed by freeze-drying. They revealed that a highly orientated porous structure of the GAs can be formed when the graphene content is higher than a threshold value, whereas the GAs fabricated with low-concentration GO precursors present an isotropic structure [176]. Yao et al. found that strong alkalis (e.g., KOH) can facilitate the formation of GO liquid crystals even at low concentrations, and the as-formed highly ordered microstructure can be inherited to the ultimate GAs upon applying hydrothermal reaction followed by freeze-drying (Fig. 9b, c) [175].

The orientation of GO liquid crystals can be further tuned by creating shear flow during the processing processes. For example, in the filtration process of graphene dispersion, the orientation direction of graphene sheets is perpendicular to the direction of the liquid flow [177, 178], while in wet spinning, the orientation direction of graphene sheets is parallel to the flow direction [179]. Li et al. reported an interesting method to tune the orientation of GO sheets by creating tilted flowing. They found that repeatedly tilting the mold containing pre-formed GO liquid crystals can impel the graphene liquid crystals to possess a long-range order through flowing and an anisotropic GA was obtained after proper reduction and drying, in which the alignment of RGO sheets along the flow direction can be observed. The thermal conductivity of the GA/paraffin composite along the graphene orientation direction reached 1.2 W m^−1^ K^−1^, which is nearly 3 times of the thermal conductivity perpendicular to the orientation direction of graphene sheets [180]. In another work reported by the same group, GO liquid crystals were loaded into a syringe and a more uniform sheet alignment was achieved by moving the pistons to generate shear forces [181]. Similarly, experimental results show that the alignment of graphene sheets along the flow direction in nozzle can be achieved by the shear stress during 3D printing process, resulting in an anisotropic porous structure in the filaments after freeze-drying [182, 183]. Some experimental studies confirm that the heat flow can also affect the orientation of GO liquid crystals. Huang et al. reported that creating temperature gradients in the GO dispersion by directional heating during the hydrothermal process can facilitate the alignment of GO sheets and lead to the formation of anisotropic GAs after freeze-drying and microwave treatments. As a result, the thermal conductivity along the alignment direction of the anisotropic GA/paraffin composite can reach 1.074 W m^−1^ K^−1^ at an extremely low graphene loading of 0.32 vol% [162]. Moreover, other methods such as adding polymer components or adjusting the solvent polarity are also effective in affecting the orientation of graphene sheets during the self-assembly process, resulting in GAs with well-arranged or layered microstructures [184–186].

### Directional Freeze-Casting Method

**Fig. 10:**
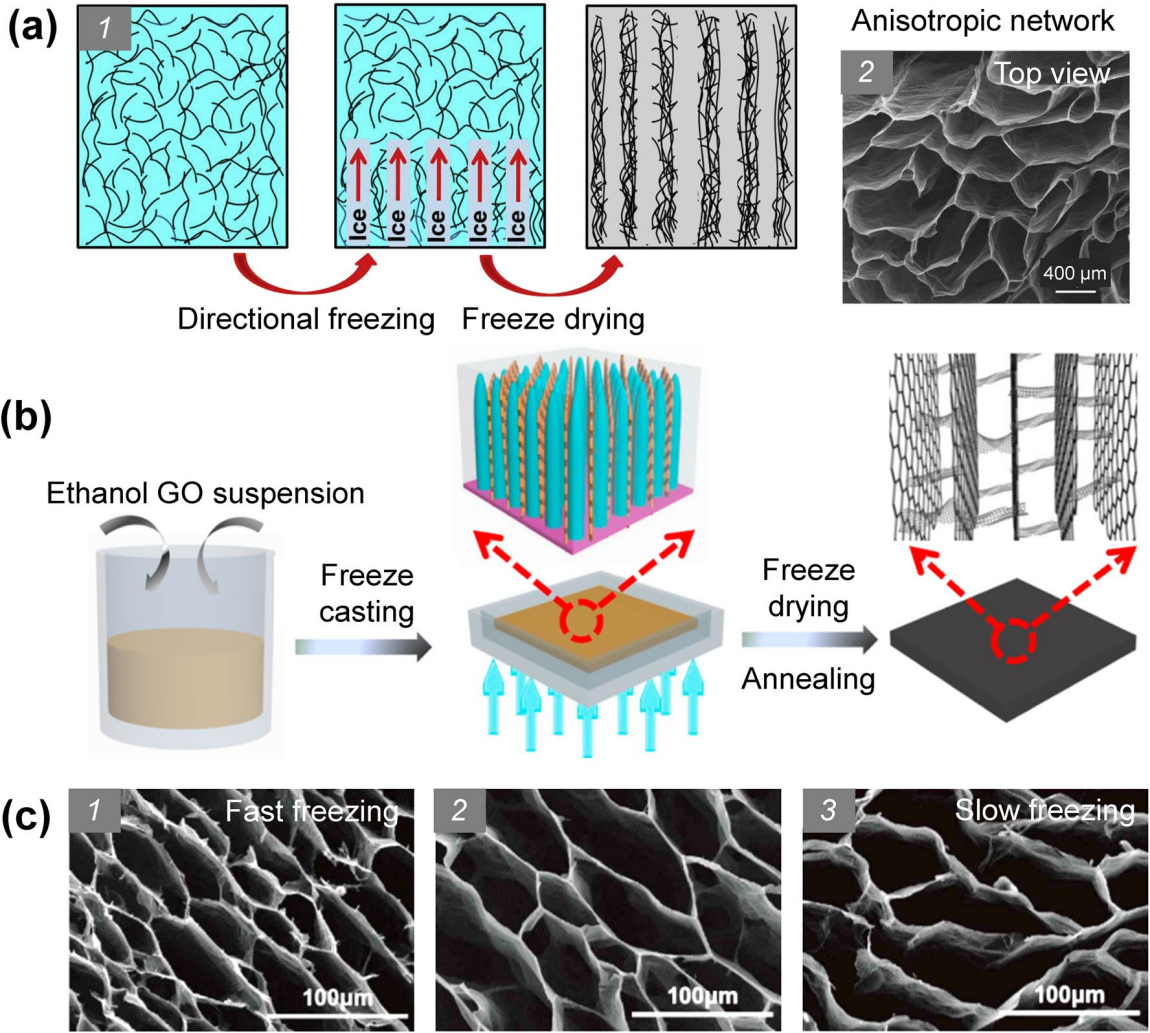
**a** Schematic of the microscopic principle of directional freezing [189]. **b** Schematic of directional freezing of GO suspension and GA structure [192]. **c** Top-view SEM images of vertically aligned graphene networks fabricated with freeze-casting at different freezing rate and subsequent freeze-drying [40]

Directional freeze-casting is another simple yet effective method for fabricating graphene networks with orientated porous structures, which enables precise structural controllability, easy scalability, and versatility [187, 188]. This technique is typically applied on wet graphene systems such as GHs and GO dispersions [189, 190]. By applying a temperature gradient in a GO/graphene suspension or GH during the freezing process, ice crystals could grow along the direction of the temperature gradient while excluding the graphene sheets, leading to close packing of graphene in the gap between ice crystals and the formation of anisotropic graphene walls. After freeze-drying, anisotropic GAs with highly oriented porous structures can be obtained (Fig. 10) [40, 120, 189, 191, 192].

Using the directional freeze-casting method to construct anisotropic graphene structures has been widely demonstrated for fabricating high-performance thermally conductive materials in recent advancements. For example, Lian et al. fabricated an anisotropic GA with GO liquid crystal dispersion as the precursor. After backfilling with epoxy, the composite exhibited a through-plane thermal conductivity of 2.13 W m^−1^ K^−1^ at a graphene loading of 0.92 vol% [60]. It is worth noting that this method is very versatile and works well for various precursor systems with a wide range of graphene concentrations. In particular, more ordered structures can be generated by using GHs as precursors because the partially reduced GO sheets in the GHs are less hydrophilic and show considerable repulsive forces to the ice crystals [120, 193]. For example, Li et al. used a GH as the precursor to obtain a GA with highly anisotropic structures by the directional freeze-casting method and the resulting epoxy composite exhibited an excellent vertical thermal conductivity of 6.57 W m^−1^ K^−1^ at an ultralow graphene loading of 0.75 vol% [40].

**Fig. 11:**
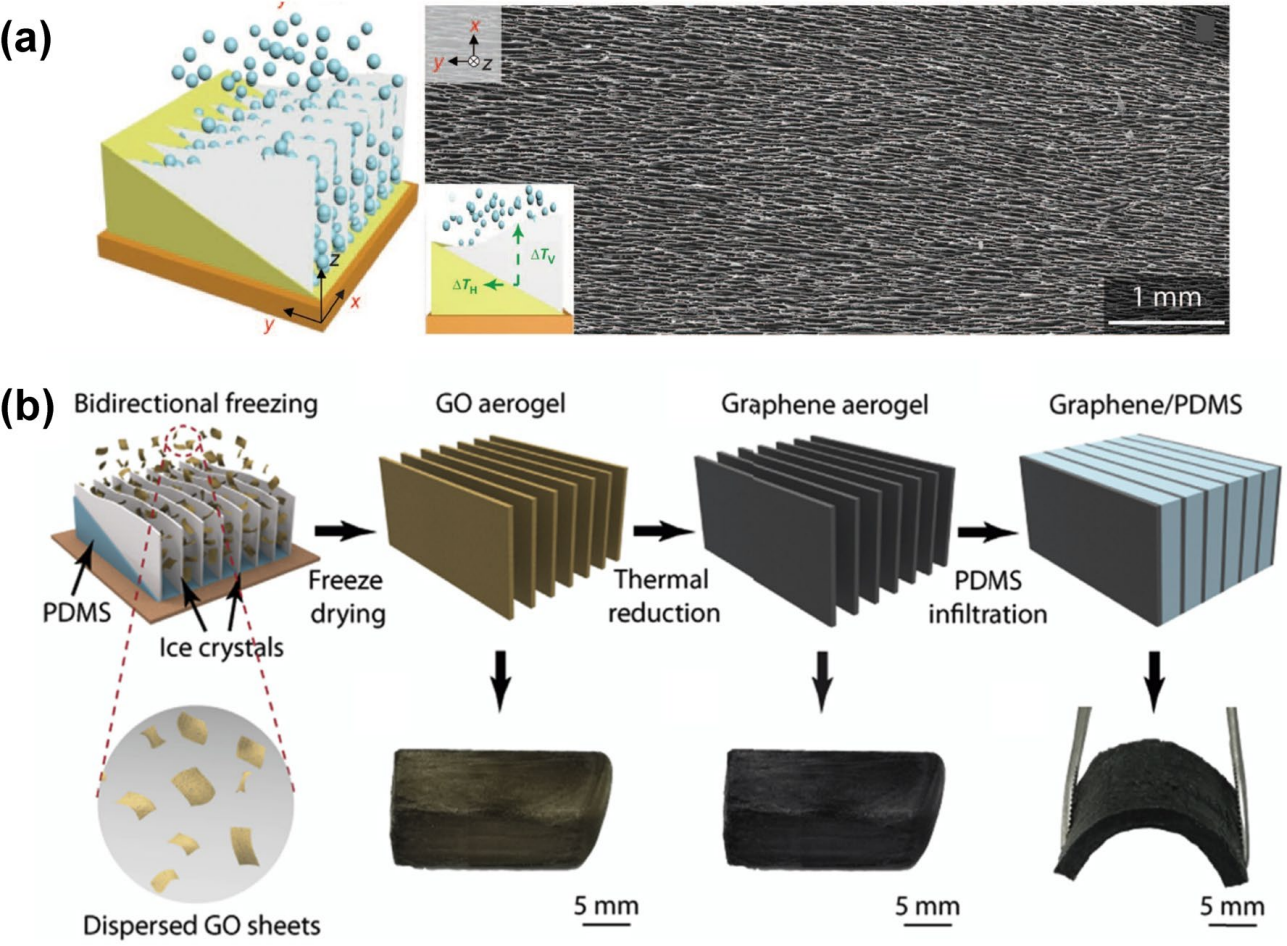
Schemes of bidirectional freezing techniques and resulting scaffolds [198, 199]

The most significant advantage of using the directional freeze-casting method to fabricate graphene 3D networks is its high controllability on the alignment of graphene sheets. Through rational design of the freeze-casting process, many novel graphene 3D structures can be easily formed [194–197]. For example, bi-directionally freeze-casting method has been used to fabricate GAs with unique lamellar structures, which relies on modification of the mold with a PDMS wedge as the spacer (Fig. 11) [198, 199]. As shown in Fig. 11a, in addition to the temperature gradient along the Z direction, the existence of the PDMS wedge can generate a temperature gradient along the Y direction, ensuring that the ice crystals can grow along two directions simultaneously to form the unique layered structures [199]. Liu et al. fabricated a high-quality graphene network with a layered porous structure from a poly(amic acid) salts (PAAS)/GO suspension by bi-directional freeze-casting. The conductive network composed of highly aligned and closely stacked graphene lamellae endowed the resultant epoxy composite with an excellent through-plane thermal conductivity of 20.0 W m^−1^ K^−1^ at a low filler loading of 2.30 vol% [70].

**Fig. 12:**
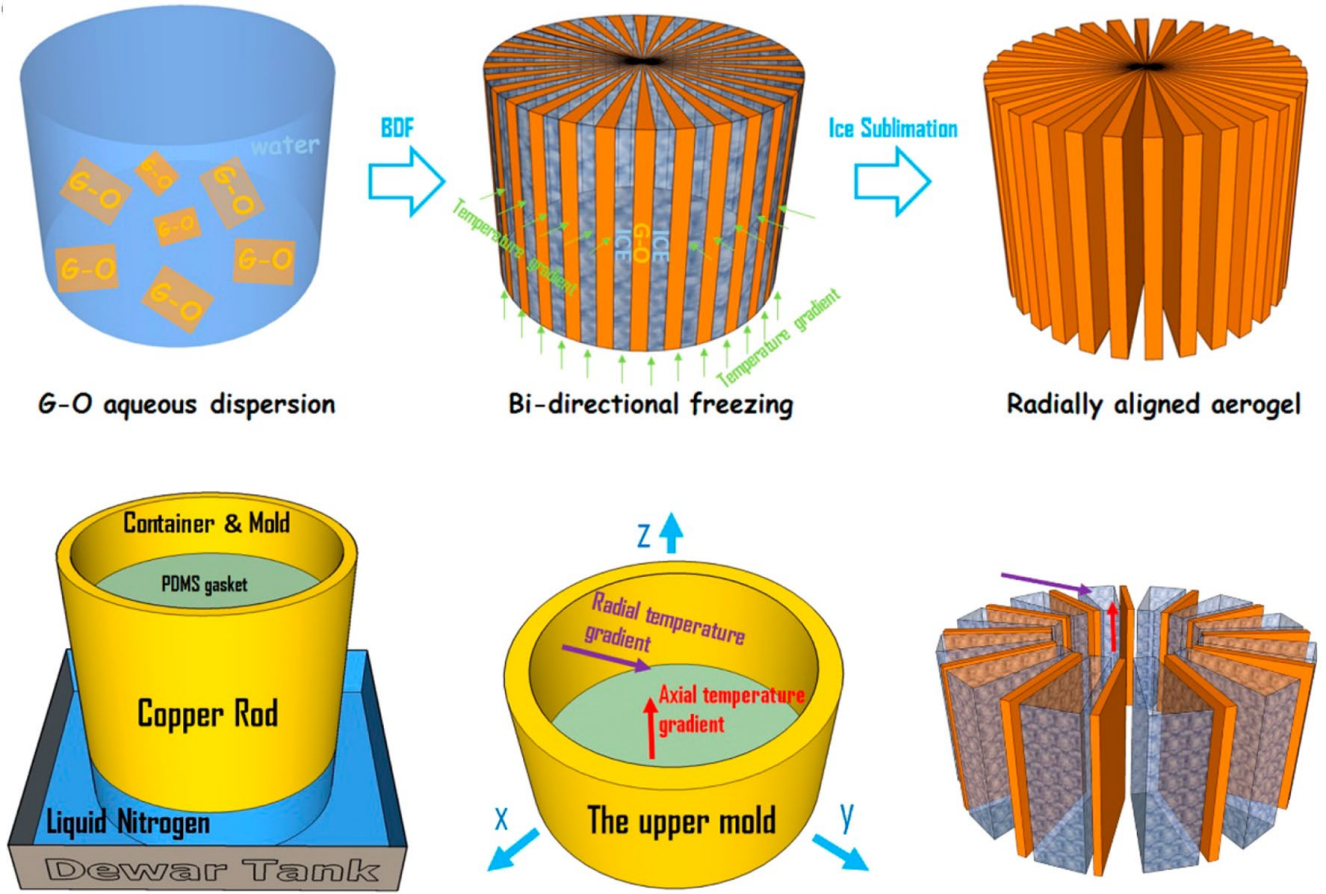
Scheme of the fabrication process of radiating GA [200]

In addition to the layered structures, radially aligned structures of graphene networks can be created by a modified directional freeze-casting method, as schematically shown in Fig. 12 [200]. During the freezing process, multi-directional temperature gradients are formed and ice crystals can grow radially, which can guide the alignment of graphene sheet to form a network with a corresponding radiating structure [200]. Bo et al. made further improvements on the basis of this structure by growing tree-leaf-like graphene nano-fins on the GA skeleton surface via CVD, which can provide additional thermal pathways and significantly reduce the boundary thermal resistance [61]. More importantly, recent progress has shown that the directional freeze-casting method can be combined with advanced manufacturing techniques, such as 3D printing, to realize the fine control on the hierarchical structures of the printed graphene materials, demonstrating excellent manufacturing flexibility and versatility [201].

**Fig. 13:**
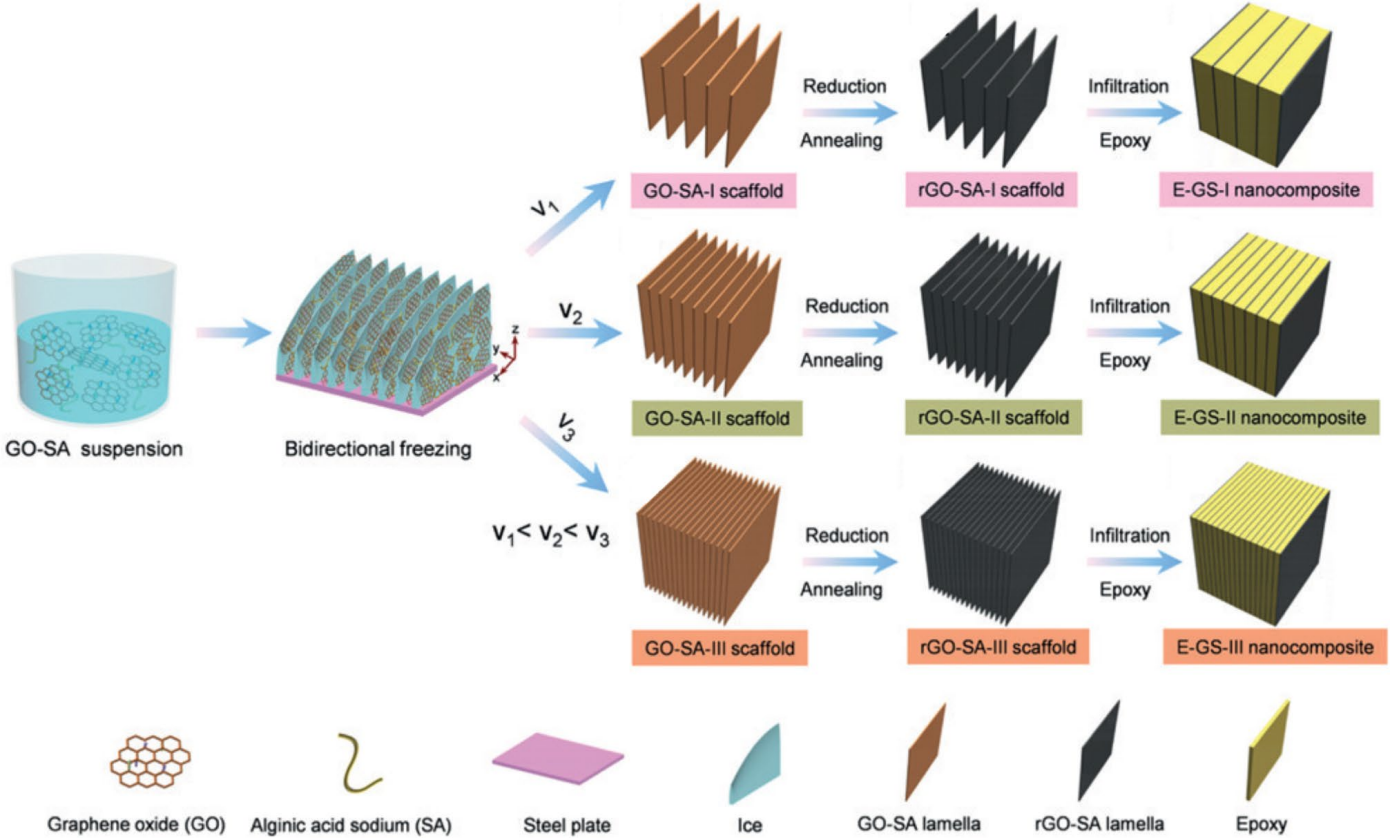
Effect of freezing rate on GA layer spacing [207]

Another advantage of the directional freeze-casting method is that the pore size, pore wall thickness, and layer spacing of the as-fabricated graphene networks can be tuned by adjusting the freezing parameters [202–205]. For example, Zhou et al. demonstrated that lower freezing rates could lead to larger pore sizes of the final graphene cellular network [206]. In another research, Huang et al. observed that the layer thickness and spacing distance can be reduced for the layered graphene network when higher freezing rate is applied during the bi-directional freeze-casting process (Fig. 13) [207]. Additionally, it has been confirmed that the pore size or layer spacing can be adjusted by tuning the concentration of the GO dispersion as the precursor [208–210]. Typically, using precursors with higher concentrations results in denser graphene networks [211]. Thanks to the high controllability and versatility, the directional freeze-casting method shows great potential for generating 3D graphene networks to possess tunable structures, ensuring the fine control of the functionality and conductive behaviors of the resulting polymer composites.

### Anisotropic Template-Assisted Assembly Method

**Fig. 14:**
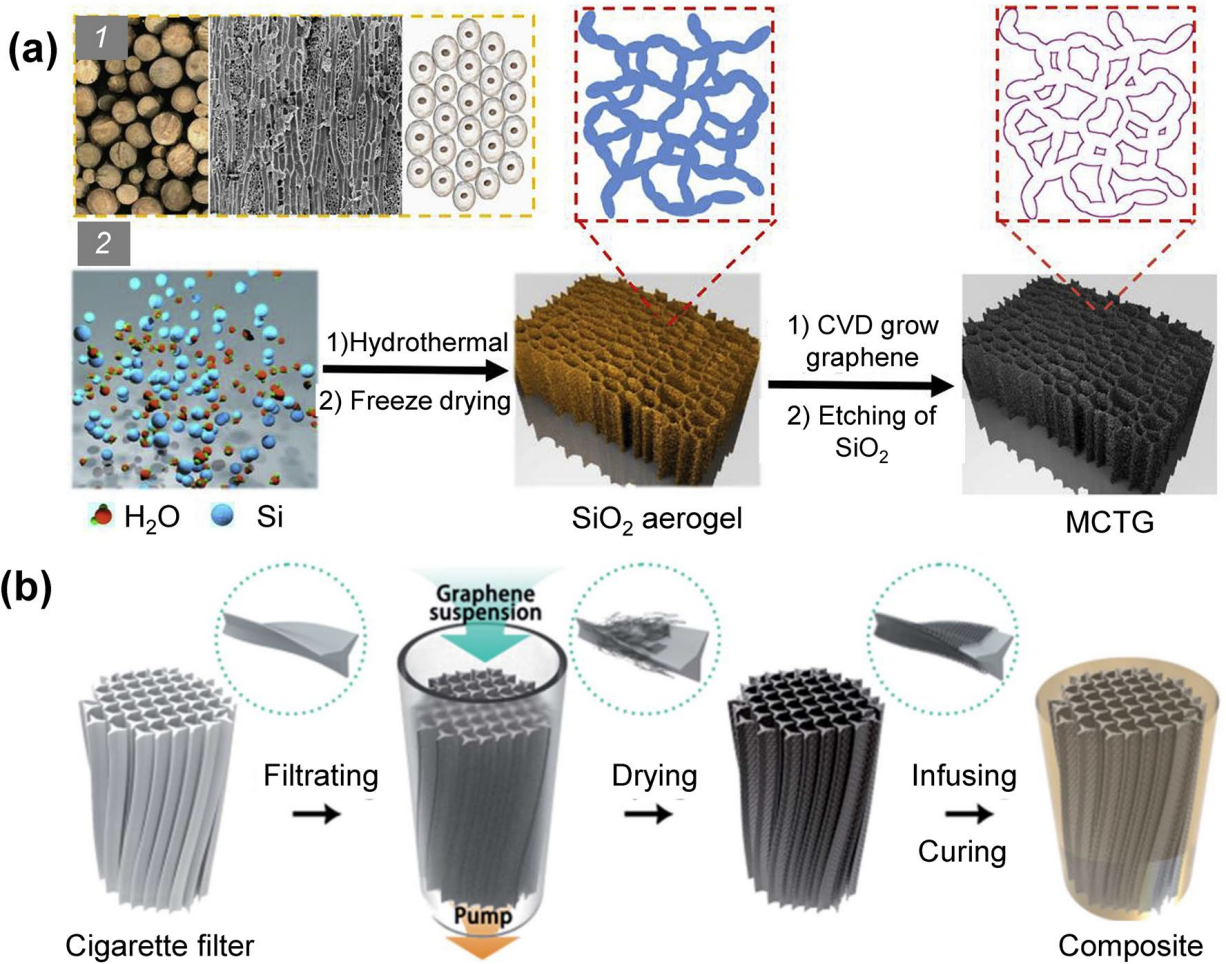
Schematic illustrations of anisotropic graphene 3D networks prepared by template method and their polymer composites [212, 213]

The template method allows the materials to inherit the structure of the templates, and therefore, anisotropic graphene networks can be obtained by depositing graphene on the skeleton surface of the templates with an anisotropic porous structure (Fig. 14) [212, 213]. SiO_2_ and Ni foams are commonly used anisotropic templates for materials that can be deposited by CVD method [212, 214]. Anisotropic 3D tubular graphene networks were fabricated by growing graphene layers with CVD on a home-made mesoporous SiO_2_ template, which can be removed later by hydrofluoric acid (HF) solution [215, 216]. Shen et al. prepared an anisotropic template by compressing the stacked Ni foam, and a corresponding 3D graphene network with multilayer oriented structure was generated by CVD approach. This unique graphene network endowed its epoxy composite with an in-plane thermal conductivity of 8.8 W m^−1^ K^−1^ at a filler loading of 8.3 wt% [217]. Some naturally occurring porous materials (e.g., woods with vertically aligned micro-channels) can also serve as templates for creating anisotropic graphene networks [218–220]. For example, a GO suspension can be filled into the porous wood-based template, and subsequently a 3D graphene/carbon network with highly aligned pores can be obtained by freeze-drying and thermal annealing [221, 222]. Other porous cellulose materials, such as the waste cigarette filters, which show affinity with graphene components, have also been used as templates for preparing anisotropic graphene networks. These templates can absorb GO dispersions and finally anisotropic carbon scaffolds coated with graphene sheets can be obtained after drying and annealing [223]. Liu et al. fabricated an anisotropic graphene network by using a cigarette filter template and converted it into a thermally conductive composite by impregnating the network with epoxy resin (Fig. 14b). The composite showed an anisotropic conductive behavior with a vertical thermal conductivity of 1.2 W m^−1^ K^−1^ [213].

### Compaction and Rolling Processes

**Fig. 15:**
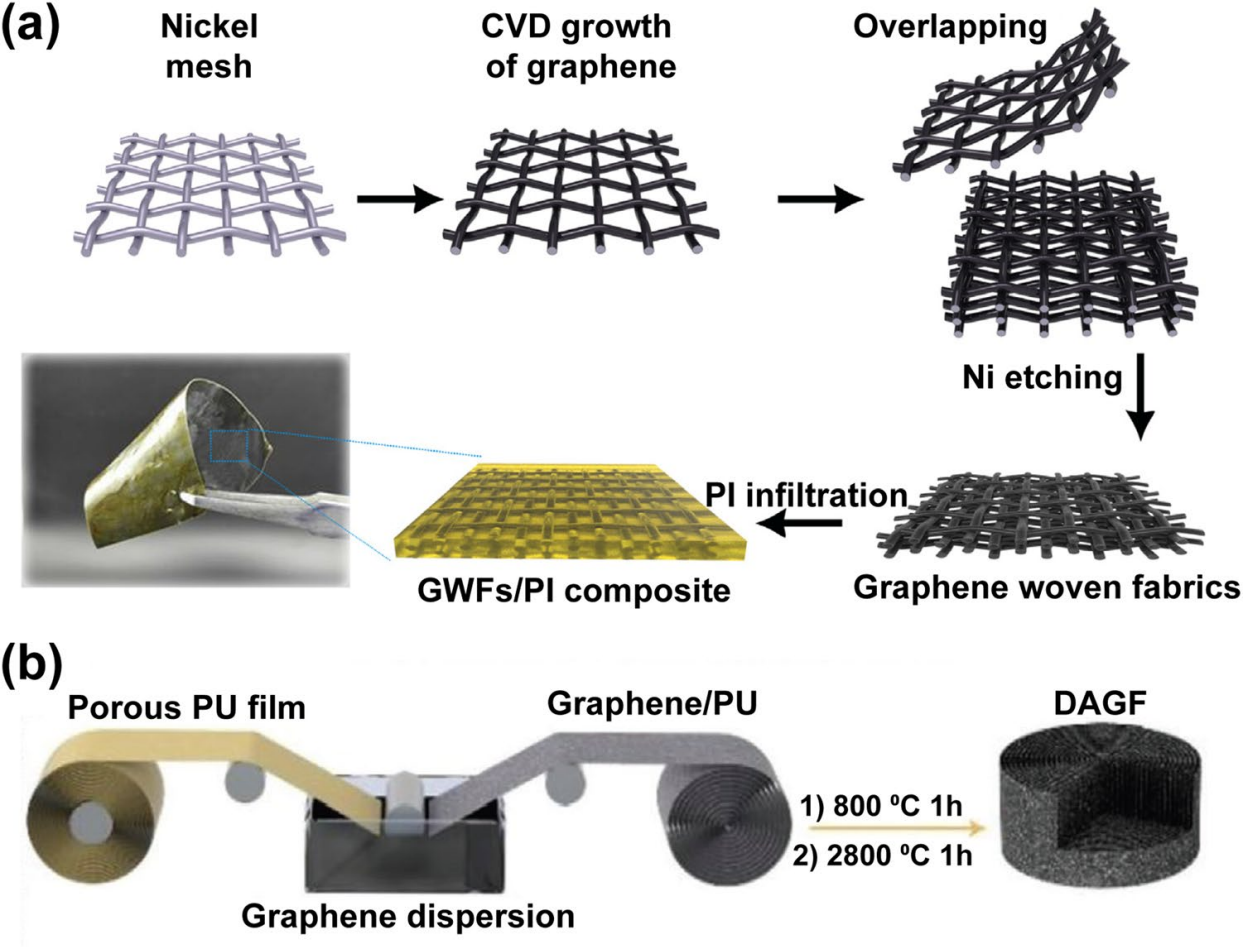
The application of **a** compaction [116] and **b** rolling [115] processing in the fabrication of anisotropic graphene networks and their composites

The anisotropy of graphene networks can be realized by post-processing, such as stacking, compaction [224] and rolling [225]. Gong et al. deposited graphene on the surface of Ni meshes by a CVD method and prepared graphene woven fabrics after the removal of the Ni template. An anisotropic graphene structure was obtained by stacking these graphene woven fabrics layer by layer (Fig. 15a), which endows the PI composite with an in-plane thermal conductivity of 3.73 W m^−1^ K^−1^ at a graphene content of 12 wt% [116]. In another study, a hollow vertically aligned graphene tube (VAGT) with macro-anisotropic properties was obtained by rolling up graphene/Ni composite fabric followed by cutting and removing the Ni skeleton. After being infiltrated with PDMS, the composite showed a thermal conductivity of 1.7 W m^−1^ K^−1^ at the graphene loading of 4.5 wt% [226]. Dai et al. used a roller equipment to stretch and roll up the porous PU-graphene film to get a large-scale monolith, in which a vertically aligned structure is formed (Fig. 15b). After graphitization and infiltration with epoxy resin, the resultant composite can deliver an unprecedented thermal conductivity of 62.4 W m^−1^ K^−1^ [115]. Rolling 2D graphene materials into 3D vertically aligned structure represents a flexible and efficient alternative to create anisotropic conducting networks. Commercially available graphene films can also be rolled up and infiltrated with polymers to fabricate high-performance thermally conductive composites [227].

In summary, designing anisotropic graphene conduction networks in composites can greatly take advantage of the intrinsic in-plane thermal conductivity of graphene sheets and enable highly efficient heat transfer along selected directions, which is very promising for thermal management applications. However, creating anisotropic graphene networks requires complicated synthesis processes, which might limit mass production. Thus, further efforts are still needed to explore more efficient and cost-effective ways, ensuring their future practical applications.

## Preconstruction of Hybrid Graphene Networks and Their Conductive Composites

Although pure graphene networks have great potentials in forming thermally conductive pathways for polymer composites, their low density and relatively weak sheet interconnections would inevitably hinder the further improvement in thermal conductivity of their polymer composites. Thus, functional additives, especially commercially available thermally conductive materials, such as GNPs [9, 77, 228], boron nitride (BN) nanoplatelets [229–231], carbon nanotubes (CNTs) [62, 232], carbon fibers [233], cellulose nanocrystals [234], copper nanowires [235], and silicon carbide nanowires [236], are usually incorporated into the graphene networks to obtain hybrid graphene networks for enhancing the structural robustness and thermally conductive properties.

**Fig. 16:**
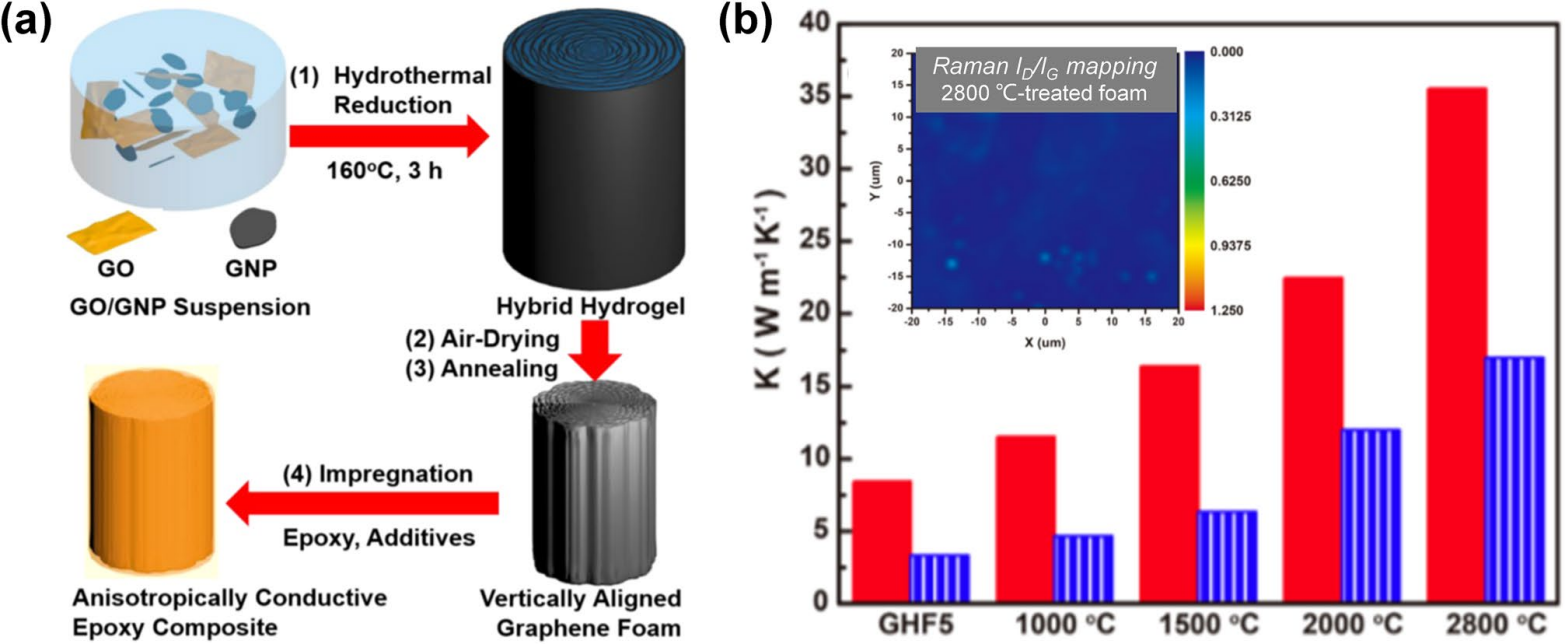
**a** Schematic illustrating the fabrication of conductive epoxy composite with vertically aligned RGO/GNP hybrid foam. **b** Through-plane conductivity (red column) and in-plane conductivity (blue column) of the epoxy composite containing the vertically aligned RGO/GNP hybrid foam annealed at different temperatures (inset: Raman *I*_D_/*I*_G_ mapping) [41]

Commercial GNPs suffer from the poor processability and easy aggregation when serving as fillers. However, GNPs have higher thermal conductivities than the RGO sheets because of their low-defect feature. To improve the thermal conductivity of graphene networks and their polymer composites, GNPs can be added into the graphene networks to form 3D hybrid networks. The graphene network serves as not only a main thermally conductive network but also a supporting framework for accommodating the GNPs and preventing their aggregation, while the presence of the GNPs can prevent excessive shrinkage of the graphene network during the fabrication process, reflecting a favorable synergy effect [237]. An et al. fabricated highly anisotropic 3D RGO/GNP hybrid networks by hydrothermal treatment of an aqueous suspension containing GNPs, GO, polyvinylpyrrolidone (PVP) and potassium hydroxide (KOH) [41]. PVP was used to facilitate the dispersion of GNPs while the KOH can restore the conjugated structures of GO sheets to induce their orderly alignment during the hydrothermal treatment. With the presence of the GNPs, the as-prepared hybrid hydrogel exhibited minimal volume shrinkage even after air-drying followed by the high-temperature annealing at 2800 °C (Fig. 16). The epoxy composite with this hybrid network showed a through-plane thermal conductivity of 35.5 W m^−1^ K^−1^ at the graphene content of 19.0 vol%, much higher than that of the composite fabricated with pristine GAs without the adding of GNPs [41]. Another strategy to incorporate GNPs into the graphene network is to infill pre-fabricated GAs with GNP dispersions. After drying to remove the solvents, the GNPs can be evenly distributed in the GA framework, which improves the continuity of the thermally conducting pathways. In particular, polymer precursors can be mixed with the GNPs dispersion and infiltrated into the GFs, and corresponding composites can be formed after polymerization or thermal curing. For example, the GF/GNP/poly(1,1-difluoroethylene) (PVDF) composite fabricated using this protocol exhibited a thermal conductivity of 6.32 W m^−1^ K^−1^ [117].

**Fig. 17:**
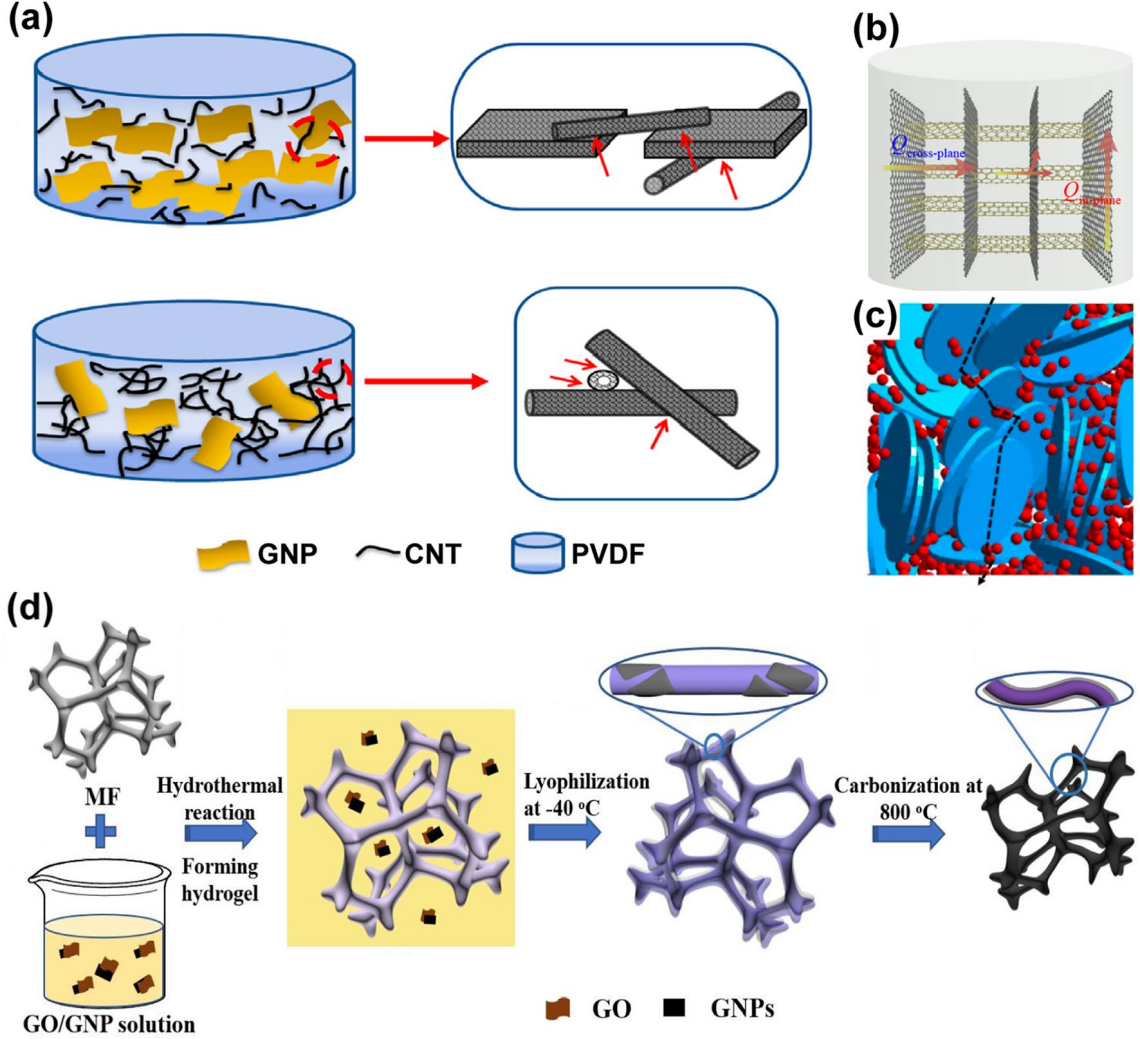
**a, b** Schematic illustrations of synergies of GNPs and CNTs on thermal conduction [62, 251]. **c** Schematic illustrations of synergies of GNPs (blue slices) and BN (red spheres) [65]. **d** Schematic illustrating the preparation of graphene/MF foam and the derived graphene/carbon foam [149]

BN nanoplatelets are also ideal fillers for fabricating thermally conductive polymer composites because of their 2D layered structure, excellent chemical stability, and extraordinary anisotropic thermal conductivity [238–240]. Meanwhile, the strong van der Waals forces allow the GO or RGO networks to have sufficient binding interactions with BN, ensuring integrity of the as-prepared hybrid networks [241, 242]. Therefore, BN nanoplatelets have been used to enhance thermal conductivity of graphene/polymer composites [204]. To improve solution processability and dispersibility of BN, surface functionalization is required. For example, the modification of BN with 3-aminopropyltriethoxysilane (APTES) can endow BN with a positively charged surface, resulting in a more homogeneous GO/BN dispersion, and the modified BN with hydrophilic amino and hydroxyl groups can interact better with the negatively charged GO sheets due to the electrostatic interactions [243, 244]. An et al. synthesized a RGO/BN hybrid aerogel by hydrothermal treatment of an aqueous GO/BN dispersion. The GO provides excellent self-assembly capability to form an interconnected network, where the BN sheets can be evenly distributed in and contribute to the formation of a denser network for more efficient thermal conduction paths. As a result, the corresponding epoxy composite exhibited a very high through-plane thermal conductivity of 11.01 W m^−1^ K^−1^ [229]. Shao et al., found that adding of 1.6 wt% of BN nanoplatelets into the graphene/PA6 composite can enhance the thermal conductivity by 87.6% [245]. It has been elucidated that the synergy between BN nanoplatelets and graphene nanoplatelets is based on three aspects: (1) the BN nanoplatelets can fill in the voids between the graphene sheets, which improves the continuity of the thermally conducting network; (2) the interactions between graphene and BN can result in compactly stacked structure, which significantly reduces the interfacial thermal resistance; and (3) the smooth surface of small BN nanoplatelets with high aspect ratio can minimize the geometric contribution to the thermal interface resistance between fillers (Fig. 17c) [65, 246].

Additionally, CNTs are 1D carbon nanomaterials with a hollow cylindrical fiber-like structure, performing excellent thermal conductivity and mechanical properties [247, 248]. The incorporation of CNTs into graphene networks can form a secondary CNT network covered on the cell wall of the graphene skeleton, which significantly enhances the thermal transfer between graphene sheets while strengthening the structure, ensuring the formation of a more efficient thermally conducting network (Fig. 17a) [249–251]. Liang et al. synthesized an anisotropic RGO/SWCNT hybrid hydrogel by hydrothermal treatment of an aqueous suspension of GO and single-wall carbon nanotubes (SWCNTs) and converted it into a highly conductive network by freeze-drying and subsequent high-temperature annealing. As schematically shown in Fig. 17b, the SWCNTs in the network effectively bridged the graphene sheets [62]. In addition, Liang et al. used the SWCNTs functionalized with hydroxyl group to enhance the coupling and bonding interactions between the conducting fillers and the epoxy matrix, resulting in a much lower interface thermal resistance. The thermal conductivity of the RGO/SWCNT/epoxy composite is 4 times higher than that of pure epoxy resin when the filler content is kept at 3.65 vol% [62]. Since both the graphene and CNT can be synthesized by CVD methods, an alternative method is proposed to generate graphene/CNT hybrid foams by designing a two-step CVD process with Ni foam as the template [252].

Cellulose nanofibers (CNFs) are usually incorporated into the graphene networks to improve the connectivity between GNPs while strengthening the resulting graphene-functionalized PCMs [253–255]. The cellulose/GNP network can not only accommodate the phase change material of PEG but also impart the PEG with enhanced thermal conductivity, excellent shape stability and mechanical properties [68, 256]. In another study, Wang et al. dispersed CNFs into graphene dispersions to improve the dispersibility of the graphene sheets and modify the interactions between the graphene and the template, so that the graphene sheets can be absorbed more efficiently on the template to form a highly continuous network [257, 258]. Other graphene hybrid networks, such as graphene/carbon foams, can be prepared by incorporating polymers (e.g., melamine) as carbon sources (Fig. 17d) [149, 259–261]. For example, graphene-coated hybrid carbon networks with a unique core–shell structure were obtained by annealing of the GO/melamine composite foams prepared by dip-coating [262, 263].

## Applications

**Fig. 18:**
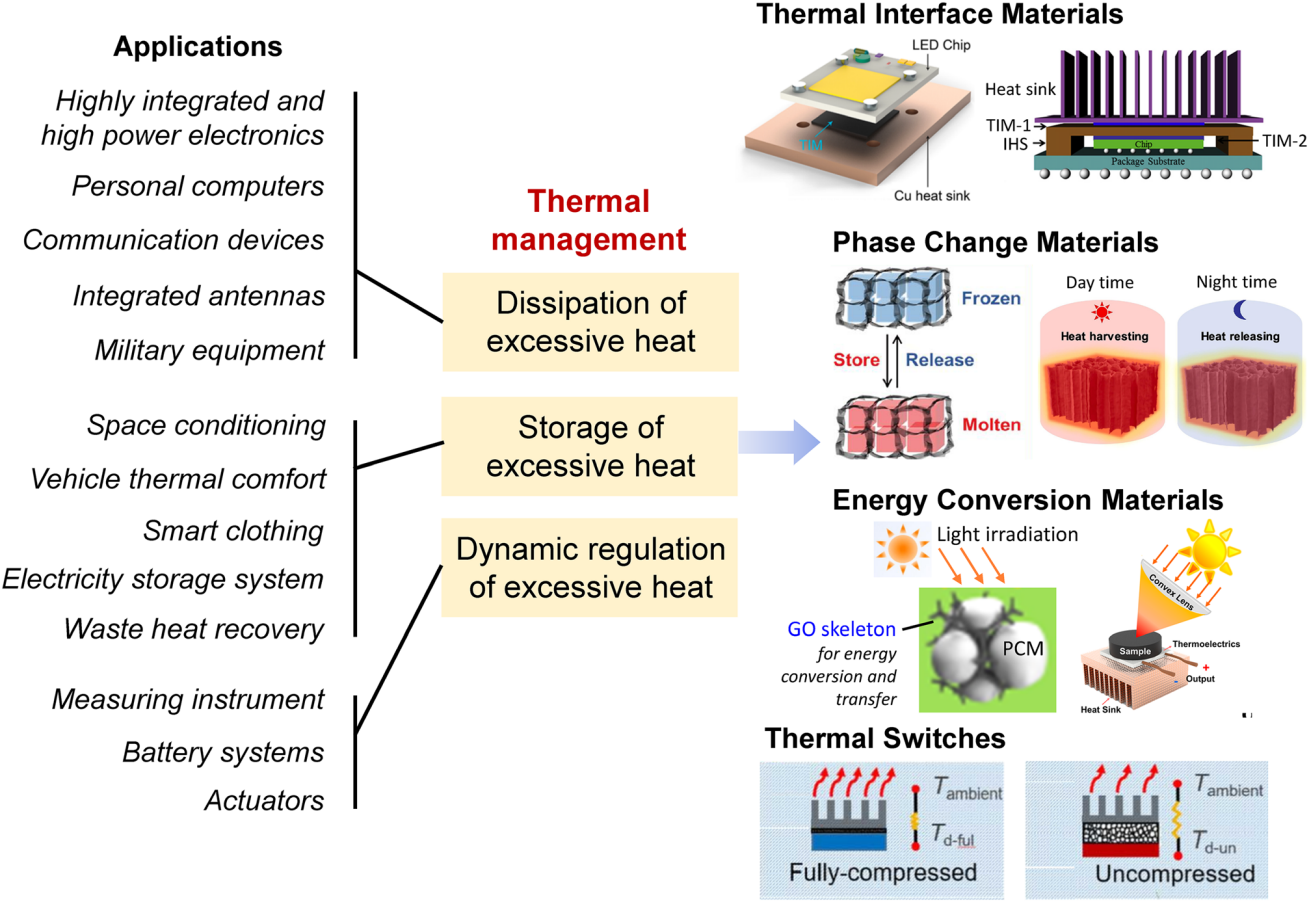
Situations where efficient thermal management of excessive heat is needed and thermally conductive polymer composites containing 3D graphene networks for various applications: TIMs [6, 45], PCMs [287, 288], energy conversion materials [283, 289] and thermal switches [269]

Thermally conductive graphene/polymer composites are highly promising for applications where efficient heat dissipation and thermal management are needed [1, 118, 264–266]. For example, electrical devices (e.g., high-frequency communication equipment) generate a lot of heat during operation and the excessive heat should be dissipated or transferred away timely to avoid overheating and ensure proper functions of the devices. To tackle the overheating problem, TIMs are urgently required to improve the heat transfer between the target devices and the heat sink and promote the heat dissipation [45, 70]. In other situations (e.g., battery system and space conditioning), the system temperature should be kept within a certain range and dynamic thermal management by PCMs or thermal switches are typically needed. PCMs can reduce temperature variation by absorbing and releasing heat via the phase change processes, and thermal switches can continuously tune the thermal transfer/dissipation behaviors by varying their own thermal conduction properties [118, 267–269] (Fig. 18). Compared with conventional manufacturing methods of graphene/polymer composites, the pre-construction of graphene networks followed by backfilling polymers enables more controllability and flexibility for fabricating high-performance thermally conductive polymer composites and would offer new opportunities to boost their applications for efficient thermal management.

### Thermal Interface Materials

TIMs are a kind of materials that are located in between two components to reduce thermal resistance between them for preventing overheating of the powerful devices by efficiently transferring heat from heat-producing devices (e.g., central processing unit) to heat-dissipating components (e.g., heat sink). In addition to high thermal conductivity, an ideal TIM should also possess satisfactory mechanical properties and long-term stability, which can prevent performance failure during operation [60, 270]. Epoxy is a typical matrix used for fabricating TIMs because of its favorable mechanical properties and high thermal/chemical resistances. Thus, high-performance TIMs can be fabricated by pre-constructing highly conductive graphene networks and subsequent backfilling and curing of epoxy resins. Li et al. synthesized an anisotropic GA/epoxy composite with a vertical thermal conductivity of 2.69 W m^−1^ K^−1^ at an extremely low graphene content of 1.11 vol%, showing a great potential as TIMs [120]. Hou et al. infiltrated epoxy resin into a prepared graphene skeleton, and the horizontal thermal conductivity of the graphene/epoxy composite was 55 times higher than that of pure epoxy resin at a low graphene loading of 5.5 wt%, which was suitable for being used as heat spreading materials [271]. Liu et al. inserted an anisotropic epoxy/graphene TIM in between the light-emitting diode (LED) chip and the copper plate for heat dissipation, and the results showed that the stabilized surface temperature of the LED chip is 13.2 °C lower than that of the chip with commercial silicone rubber-based TIM [70]. Although significant progresses have been made in designing graphene/polymer TIMs, current studies focused primarily on enhancing the thermal conduction performances of materials, while technical issues related to practical applications (e.g., thermal expansion during operation and installation difficulties) are less considered. Further effort is also needed to develop TIMs with favorable application flexibility.

### Phase Change Composites and Photothermal Conversion Materials

PCM promises sustainable energy conversion and can provide useful thermal management by absorbing and releasing thermal energy during its phase changing process. An ideal PCM should have high thermal conductivity, high heat of fusion, high specific heat, proper density, high melting point, high shape stability, and long-term reliability during repeated cycling. However, most of conventional pristine PCMs (e.g., paraffin wax) suffer from problems of low thermal conductivity and poor shape stability, which hinder the wide applications of these PCMs. Incorporating graphene sheets as the thermally conductive fillers into pristine PCMs is promising for enhancing their thermal conduction and comprehensive performances [272–274]. The graphene network in the PCMs can not only enhance the heat transport properties but also serve as a supporting framework to improve structural robustness of the PCMs, which enables high shape stability even at temperatures above the melting points of PCMs. For example, Liu et al. reported that the combination of an air-dried RGO/GNP hybrid network with 1-octadecanol PCM can result in a great enhancement of thermal conductivity from 0.21 W m^−1^ K^−1^ for the 1-octadecanol to 9.50 W m^−1^ K^−1^ and the resultant composite has a high melting enthalpy of 196.2 J g^−1^ [267]. Notably, typical pristine PCMs, such as PEG, paraffin wax, n-hexadecane, and 1-octadecanol, possess favorable fluidity at elevated temperatures. Thus, high-performance phase change composites (PCCs) can be produced by backfilling the melted PCMs into the pre-constructed graphene 3D networks. Optimization of PCCs can be achieved by structural/compositional designs of graphene networks. The embedded graphene skeletons can effectively prevent the leakage of PCMs due to the capillary forces, which significantly improves the shape stability of PCCs and ensures outstanding durability for practical applications [267, 275–277].

With the rapid industrial development, the energy crisis and related water pollution problems are becoming increasingly serious. Different from non-renewable fossil resources, solar energy is considered a kind of green energy resource. Obviously, efficient conversion of solar energy to usable thermal energies represents one of the most promising and sustainable routes to relieve energy crisis and environmental issues [278, 279]. Because of the unique combination of excellent thermal conduction, phase-changing enthalpy, and photothermal conversion performance, graphene-functionalized PCCs are ideal for converting solar energy and storing thermal energy [280]. Xue et al. reported that the temperature of PCMs functionalized by graphene can reach 79.6 °C with a high photothermal conversion efficiency of 78% at one-sun illumination. Moreover, since the maximum temperature of the composite under solar light illumination is above the phase change temperature, the heat energy can be stored in the PCM matrix during the phase transition [257]. For real-life application scenarios, PCCs can be placed on the roof of buildings to keep temperature within a certain range. In the daytime, PCCs absorb solar energy for photothermal conversion and maintain the temperatures near the melting points. After the disappearance of sunlight in the night, the PCCs would release the heat through the phase transformation process to slower the indoor temperature drops [263]. In another application scenario, graphene-based photothermal conversion materials were connected to the thermoelectric conversion device to output electric energy, realizing successful photo-thermal-electric conversion [204, 240, 281, 282]. Cao et al. assembled a photo-thermo-electric conversion device by combining the graphene-functionalized PCC with a temperature differential power generator, which enables an output voltage of 144 mV under an illumination of 200 mW cm^−2^ [282]. Liu et al. designed a PEG-based PCC containing a radially aligned GO/BN network, which was then assembled with a thermoelectric generator to obtain a solar thermoelectric generator. Since the unique conductive GO/BN network in the PCC endows the device with rapid heat diffusion capability to reduce local thermal accumulation, a convex lens can be applied to concentrate the solar light. Under real sunlight conditions, the output voltage and the output power density of the solar thermoelectric generator can reach 251 mV and 40.28 W m^−2^, respectively [283].

### Thermal Switches

In addition to the above-mentioned applications, thermal switch is an emerging application of graphene composites, which provides dynamic thermal management to allow the devices to function well at dramatically varied conditions. A thermal switch can provide switchable regulation of the heat dissipation pathways via changing its thermally conducting properties. Thus, the materials for this application should be thermally conductive and their thermal conductivity should be sensitive to external stimuli (e.g., compressing and stretching). In addition, these materials are also required to have satisfactory mechanical properties and durability [193, 284]. For example, a compressive switch should have good elasticity and outstanding fatigue resistance, which could ensure long-term stability of the functions upon repeated compressive deformation during operation. It is reported that favorable thermal regulating function can be realized by preparing elastic thermally conductive graphene/polymer composites, such as composite foams. These elastic foams are designed to be poorly thermally conductive at the initial state, representing an “off” state. Upon compression, the conductivity increases because the conductive network is denser, leading to the formation of more efficient conducting pathways (“on” state) and the conductivity can be easily modulated by adjusting the degree of compression [285, 286]. For example, Du et al. fabricated a thermal switch based on an elastic graphene foam, which presents continuous tunability, wide tuning range, and fast response. The graphene foam switch provides a large (~ 8x) continuous adjustment of its thermal resistance from uncompressed state (“off” state) to full compression (“on” state) and can accurately stabilize the operating temperature of the target device, holding great potential for dynamic thermal management applications in electronic devices and batteries [269].

## Conclusion and Perspective

In this review, we have discussed the recent progress in improving thermally conductive properties of graphene-functionalized polymer composites with a focus on preconstruction of 3D graphene conducting networks. Achieving high thermal conductivity for polymer composites relies on the formation of highly continuous and high-quality thermal conduction network in polymer matrices, which is the key to ensuring efficient heat transfer. Conventional processing methods, such as solution mixing and melt compounding, usually suffer from the aggregation of graphene sheets during the mixing/compounding processes, and thus effective thermal conduction networks can only be formed at high graphene loadings, which would result in limited thermal conductivities, high cost, and degraded mechanical properties of the composites. In comparison, preconstruction of 3D graphene networks followed by backfilling polymers/monomers provides a promising route to fabricate thermally conductive composites with improved performances. The preformed conductive networks can be well maintained during the mixing/compounding process without producing agglomeration of graphene sheets in polymer matrices, ensuring the high thermal conductivity of the as-fabricated composites at low filler loadings while avoiding performance degradation of polymer matrices. The thermally conductive behavior of the resultant composites can be modified according to practical application scenarios by configurational/microstructural designs of preconstructed 3D networks, such as designing anisotropic conductive networks for enabling directional conduction behavior, which provides unique flexibility and versatility for composite fabrication. Although significant progress has been achieved, many challenges remain and there is still much to be developed for controllable preconstruction of graphene networks and the fabrication of their composites.

1. From the aspects of graphene networks, their thermal conduction is largely affected by the quality of the graphene building blocks. The most popular method to construct high-quality 3D graphene networks is using GO as the precursor for assembly followed by high-temperature annealing. The defect-free feature of thermally healed graphene can endow the 3D network with excellent thermal conductivity but would also lead to poor structural stability due to the poor interfacial interactions between the high-quality graphene sheets, which brings difficulty to subsequent compounding process, especially in mass production. Thus, scalable and efficient approaches to combine superb conductivities and good structural robustness into one high-quality graphene network are highly demanded.
2. Regarding the design of anisotropic networks, various methods, e.g., ice templating method, has been proved to be effective. However, the long-range order and customizability of the as-obtained graphene network still needs to be improved. Thus, some efforts should be focused on optimizing the existing methods and understanding their fundamental principles and mechanisms for achieving better controllability.
3. Although the preconstruction of 3D graphene networks can ensure the high continuity of the conductance pathways in polymer matrix, the interfacial thermal resistance between graphene and polymer is still a critical obstacle for further enhancement of thermal conductivity of the composites. More attention should be paid to this issue, making it possible to approach the upper limit of thermal conductivity of graphene-based polymer composites.
4. Most previous efforts have been focused on improving thermal conductivity of materials. However, integrating more functions (e.g., fire-retardant and self-extinguishing properties, sensing capability, electromagnetic interference shielding, and self-adhesive property) into the graphene-based polymer composites might bring more opportunities for broadening their future applications. In addition, more investigation is needed to understand the roles of graphene-based polymer composites in practical applications for expanding their commercial potentials. At present, there is still a long way to go for scalable production and commercial application of polymer composites with preconstructed graphene conductance networks. However, the development of techniques for tuning the graphene dispersion behavior in polymer matrices will continue to support the design and preparation of next-generation functional composites for thermal management applications.
